# Knockdown of Oligosaccharyltransferase Subunit Ribophorin 1 Induces Endoplasmic-Reticulum-Stress-Dependent Cell Apoptosis in Breast Cancer

**DOI:** 10.3389/fonc.2021.722624

**Published:** 2021-10-27

**Authors:** Jiajun Ding, Jiahui Xu, Qiaodan Deng, Wei Ma, Rui Zhang, Xueyan He, Suling Liu, Lixing Zhang

**Affiliations:** ^1^ Fudan University Shanghai Cancer Center & Institutes of Biomedical Sciences, Cancer Institutes, Key Laboratory of Breast Cancer in Shanghai, The Shanghai Key Laboratory of Medical Epigenetics, Key Laboratory of Medical Epigenetics and Metabolism, Shanghai Medical College, Fudan University, Shanghai, China; ^2^ Breast Surgery, Obstetrics and Gynecology Hospital of Fudan University, Shanghai, China

**Keywords:** breast cancer, ribophorin 1, oligosaccharyltransferase complex, endoplasmic reticulum stress, apoptosis

## Abstract

Ribophorin 1 (RPN1) is a major part of Oligosaccharyltransferase (OST) complex, which is vital for the N-linked glycosylation. Though it has been verified that the abnormal glycosylation is closely related to the development of breast cancer, the detail role of RPN1 in breast cancer remains unknown. In this study, we explored the public databases to investigate the relationship between the expression levels of OST subunits and the prognosis of breast cancer. Then, we focused on the function of RPN1 in breast cancer and its potential mechanisms. Our study showed that the expression of several OST subunits including RPN1, RPN2, STT3A STT3B, and DDOST were upregulated in breast cancer samples. The protein expression level of RPN1 was also upregulated in breast cancer. Higher expression of RPN1 was correlated with worse clinical features and poorer prognosis. Furthermore, knockdown of RPN1 suppressed the proliferation and invasion of breast cancer cells *in vitro* and induced cell apoptosis triggered by endoplasmic reticulum stress. Our results identified the oncogenic function of RPN1 in breast cancer, implying that RPN1 might be a potential biomarker and therapeutic target for breast cancer.

## Introduction

Breast cancer (BC) is one of the leading causes for the mortality of women all over the world. It accounted for 24.2% of the 8.6 million new cases of female cancer and 15.0% of 4.2 million cancer-related deaths in women worldwide in 2018 (1). BC is a complex and heterogeneous disease. Four major intrinsic molecular subtypes, which are Luminal A, Luminal B, HER2-enriched, and basal-like breast cancer (BLBC), have been identified (2). Among which, BLBC is recognized as the worst subtype due to the lack of effective treatment. Although great progress has been made in the diagnosis and treatment of BC, finding new targets for early diagnosis and treatment remains a challenge. Recently, large accessible databases like Oncomine have become efficient and economic tools for identifying targets for BC (3, 4). And they may play an important role in identifying novel genes associated with BC.

N-linked glycosylation is a vital protein modification in eukaryotic cells. Proteins are N-glycosylated in the endoplasmic reticulum lumen by Oligosaccharyltransferase (OST) complex. Although the exact structure of OST in eukaryotes is largely unknown, it has been found that OST complex consists 12 subunits, including STT3 OST complex catalytic subunit A and B (STT3A, STT3B), Ribophorin 1 (RPN1), Ribophorin 2 (RPN2), dolichyl-diphosphooligosaccharide-protein glycosyltransferase (DDOST), defender against cell death 1 (DAD1), oligosaccharyl-transferase complex subunit 4 (OST4), transmembrane protein 258 (TMEM258), oligosaccharyltransferase complex (OSTC) and keratinocyte associated protein 2 (KRTCAP2), magnesium transporter 1 (MAGT1), and tumor suppressor candidate 3 (TUSC3) (5–8).

The abnormality of OST subunits can lead to the hypoglycosylation of proteins, which account for the misfolding of proteins. The accumulation of misfolded proteins would affect the homeostasis of endoplasmic reticulum, ultimately inducing an imbalance between protein folding load and capacity. This abnormality is known as endoplasmic reticulum stress (ERS) (9), which is associated with the development and prognosis of cancers (10–13). At first, ERS initiates unfolded protein response (UPR) to improve the adaptability and reestablish the homeostasis. With the persistent ERS, the UPR could turn from a pro-survival to a pro-death response, playing a biswitch role in homeostasis maintenance (14).

RPN1, which is only found in the rough endoplasmic reticulum, facilitates the N-glycosylation by selecting the specific substrates (15). Though it is a critical subunit of OST, the association between RPN1 and cancers has rarely been reported. In this study, we analyzed the relationship between OST subunits, especially RPN1, and BC by several accessible databases, and then explored the effects of RPN1 knockdown on the proliferation, migration, and invasion of BC cells. Finally, we found that the ERS-induced cell apoptosis was responsible for the inhibition of cell proliferation and invasion after RPN1 knockdown.

## Materials and Methods

### Oncomine Database Analysis

Expression level of the OST subunits in various cancer types was retrieved from Oncomine (http://www.oncomine.org, accessed on February 28, 2019) (3). Thresholds were set as the following: p-value: 0.0001; fold change: 1.5; gene rank: top 10%; and data type: mRNA. After analyzing the mRNA expression level in different cancers, we additionally performed a meta-analysis with the providing 13 datasets, which contained 43 analyses of 3,555 samples on different kinds of BC (**Supplementary Table S1**), aiming to compare the over-expression variation of different subunits. p-value<0.01 was considered statistically significant.

### BC Gene-Expression Miner v4.5 Analysis

Bc-GenExMiner v4.5 (bcgenex.centregauducheau.fr/, accessed on August 3, 2020) was used to measure the correlation between the OST subunits and the clinicopathologic features in BC (4). P-value<0.01 was considered statistically significant. The Pearson correlation coefficient between the expression level of candidate genes and RPN1 was computed to determine the co-expressed genes of RPN1. We identified genes as the co-expressed genes of RPN1 when the Pearson correlation coefficient > 0.4.

### Survival Analysis

Kaplan-Meier Plotter (kmplot.com, accessed on March 3, 2019) was used to identify the prognostic genes among OST subunits in BC (16). We also identified the prognostic genes among OST subunits in each subtype of BC, and the subtype of BC was determined by the 2013 St Gallen criteria. The patients were divided into two groups (high expression and low expression) by the median value of gene expression level, and only the best probe for each gene was selected. The hazard ratio (HR) with 95% confidence intervals (CI) and log-rank Genes with P-value<0.05 was considered as prognostic genes.

### The Cancer Genome Atlas and Gene Expression Omnibus Database Analysis

The TCGA and GEO database (GSE42568) were used to explore the expression of RPN1 in BC tissues and normal breast tissues or para-tumor tissues. The expression level of RPN1 in each subtype was also analyzed in TCGA and GEO database (GSE47561). In addition, using the transcriptome data from TCGA, we evaluated the co-expression level between two of OST subunits by custom R scripts.

### GeneMANIA Analysis

As a prediction server for gene prioritization and predicting gene function (17), GeneMANIA database (http://genemania.org/, accessed on January 17, 2020) was used in our study to construct an interactive functional-associated network for OST subunits in terms of physical interactions, predictions, pathways, shared protein domains, co-expression, co-localization, and genetic interactions, as well as to find their functions.

### The Human Protein Atlas Database Analysis

We used the HPA (https://www.proteinatlas.org/, accessed on December 19, 2019) to explore the immunohistochemical (IHC) staining of RPN1 (18–20). The images of normal breast tissues were gotten from the TISSUE ALTAS, while the images of BC tissues were gotten from the PATHOLOGY ALTAS. Both normal breast and BC tissues were stained by antibody CAB009748.

### UALCAN Database Analysis

UALCAN (http://ualcan.path.uab.edu/, accessed on December 20, 2019) is an interactive database for analyzing cancer omics data, including TCGA data and the Clinical Proteomic Tumor Analysis Consortium (CPTAC) data (21). We used UALCAN to analyze the protein level of RPN1 in BC tissues compared to the normal breast tissues in CPTAC samples and the methylation level on the promoter region of RPN1 in TCGA samples.

### Functional Enrichment Analysis

Gene Ontology (GO) enrichment analysis for gene lists from Bc-GenExMiner v4.5 database was conducted using the R package “clusterProfiler”, “org.Hs.eg.db”, “enrichplot” (https://bioconductor.org/, accessed on January 18, 2020), and “ggplot2” (https://cran.rproject.org/web/packages/, accessed on January 18, 2020). Only the top five significant enriched GO terms were plotted.

### Cell Culture and Reagents

The human BC cell lines SUM149 and SUM159 (purchased from Asterland Bioscience, MI, USA) were confirmed without mycoplasma and then cultured in Han’s F12 medium with 5% fetal bovine serum (FBS, Thermo Fisher), 1% streptomycin/penicillin (Beyotime), 1 mg/ml hydrocortisone (Sigma-Aldrich), 10 ug/ml gentamicin (Life Technologies), and 5 mg/ml insulin (Sigma-Aldrich). All cells were incubated under 37°C with 5% CO_2_. Sodium phenylbutyrate (4-PBA) was purchased from MCE and dissolved in DMSO.

### Virus Infection and Cell Lines Construction

The effective sequences of shRNAs were bought from Sigma-Aldrich (**Supplementary Table S2**). The RPN1 knockdown lentiviruses were produced by transfecting 293T cell in the University of Michigan Vector Core Facility. SUM159 and SUM149 cells were infected in the presence of polybrene (8 ug/ml, Millipore) for 24 h, then the medium was discharged and replaced with the fresh medium. And knockdown cells were selected by Puromycin (Invitrogen) for 14 days.

### RNA Extraction and Real-Time qRT-PCR

Total RNA was extracted using Trizol (Takara) and reverse-transcribed into cDNA with the HiScript II 1st Strand cDNA Synthesis Kit (Vazyme Biotech). The primers for qRT-PCR were provided in **Supplementary Table S3**. And qRT-PCR was carried out using AceQ qPCR SYBR Green Master Mix (Vazyme Biotech) in a real-time PCR system (7300, Applied Biosystem). TATA-box binding protein (TBP) was used as a reference gene.

### MTT Assay

One thousand cells of SUM159 and 3,000 cells of SUM149 were seeded in per well of 96-well plates and cultured for 1 day for eliminating the counting error. Two hundred cells of SUM159 and 500 cells of SUM149 were did the same at the same time but cultured for 3, 5, and 7 days. Then 20 ul MTT (5 mg/ml, Biosharp) was added in each well, and the plates were incubated at 37°C for 4 h. After removing the supernatant, 100 ul DMSO was added in per well, and the optical density (OD) was measured at 490 nm with microplate reader (Elx800, BioTek). Each group had six parallel wells and was performed in triplicate.

### Colony Formation Assay

One thousand cells of SUM159 and 3,000 cells of SUM149 were seeded and cultured in six-well plates under 37°C for 2 weeks. Ten percent formaldehyde was used for fixing for 30 min, and the cell colonies were stained with 0.1% crystal violet for another 30 min. After washing and drying, the number of colonies was calculated. Each group had three parallel wells and was performed in triplicate.

### Wound Healing Test

One million cells of SUM159 were seeded in six-well plates and grew to approximately total confluence. Then the wounds were created by a 200 ul pipette tip. The wells were washed by PBS for two times, and none-serum medium was added. Wound healing within the scrape lines were then observed and photographed at 0, 6, 18, and 24 h. Each group had more than three parallel positions and was performed in triplicate.

### Invasion Assay

Transwell chambers (#3422, Corning, USA) precoated with matrigel (354234, Corning, USA) were placed in 24-well plates at 37°C for 4 h. Then 5×10^4^ cells of SUM159 were plated on chambers without serum and medium containing 5% FBS offered in the bottom well. After 36 h of incubation in normal condition, the chambers were fixed (methyl alcohol: glacial acetic acid = 3:1) and stained with 0.1% crystal violet. After washing and drying, the invaded cells were photographed for statistical analysis. Each group had three parallel wells and was performed in triplicate.

### Western Blot

Cells were lysed in RPRA buffer (Beyotime, China), and protein concentration was measured by BCA Kit (Pierce, USA). Protein samples were separated by SDS-PAGE and subsequently transferred onto PVDF membranes. Membrane was blocked in 5% de-fat milk and incubated with primary antibody at 4°C for a night and sequentially HRP-conjugated secondary antibody at room temperature for 1 h. ImageQuant LAS 4000 mini imaging system (GE, Fairfield, USA) and Western HRP Substrate (WBLUF0500, Millipore) were used in chemiluminescent detection. The antibody used in this study are as following: anti-GAPDH (M017, TransGen), anti-PERK (5683, CST), anti-IRE1α (3294, CST), anti-ATF6 (24169-1-AP, Proteintech), anti-BiP (3177, CST), anti-Bax (2774, CST), anti-Bcl-2 (4223, CST), goat anti-mouse IgG-HRP (sc-2005, Santa Cruz), and goat anti-rabbit IgG-HRP (sc-2004, Santa Cruz).

### Flow Cytometry

For apoptosis analysis, the cells were stained with annexin V-fluorescein isothiocyanate (BD) and propidium iodide (Sigma-Aldrich) for 15 min at room temperature. The stained cells were measured by flow cytometer according to the manufacturer’s instructions. For cell cycle analysis, cells were fixed in 70% ethyl alcohol at 4°C for 6 h and then stained with propidium iodide containing 1% RNase A (Takara) at 37°C for 30 min. CytoFLEX (Beckman Coulter) was used for the detection and acquisition of data, and the analysis was performed in CytoExpert software.

### Statistical Analysis

All data were present as the mean ± standard deviation. Difference between two groups was analyzed by Student t test with GraphPad Prism 6. Some results of statistical analysis were download from the websites directly. P<0.05 was considered statistically significant unless otherwise indicated.

## Results

### Correlations of Transcriptional Expression Among OST Subunits and Construction of a Protein-Protein Interaction Network

**Figure 1:**
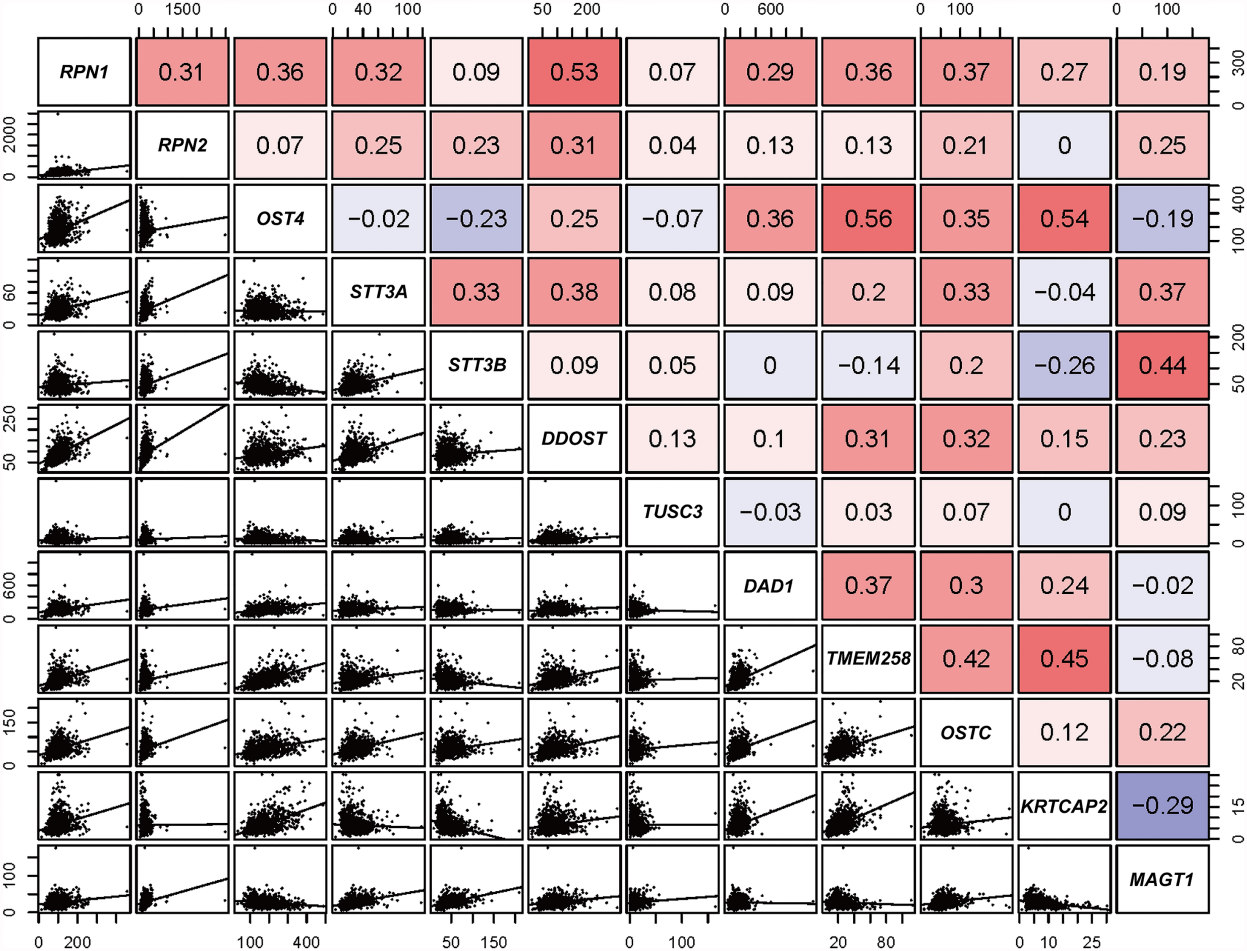
Correlation analysis of OST family members (data from TCGA). Pearson’s correlation of OST subunits. In the upper right, red and blue cells represent positive and negative relationship, respectively. In the lower left, correlation scatter diagram of the two genes is listed.

According to the transcriptional data from TCGA database, the Pearson correlations among OST subunits in BC patients were analyzed (**Figure 1**), and Pearson correlation coefficient exceeding 0.40 indicated a good correlation. It could be found that there was a significant positive correlation between RPN1 and DDOST, STT3B and MAGT1, TMEM258 and OSTC, as well as among OST4, TMEM258, and KRTCAP2.

Then, GeneMANIA database was used to construct a protein-protein interaction network for the OST subunits and to analyze their potential functions (**Supplementary Figure S1**). The 12 central nodes represented the OST subunits, and the 20 nodes surrounding represented the top 20 genes that correlated to the OST subunits in terms of physical interactions, predictions, pathways, shared protein domains, co-expression, co-localization, and genetic interactions. And further functional analysis showed that the 12 central genes we focused on were as expected greatest related to the OST complex and the function of glycosylation.

### mRNA Expression Profiles of the Subunits of OST in BC Patients

**Figure 2:**
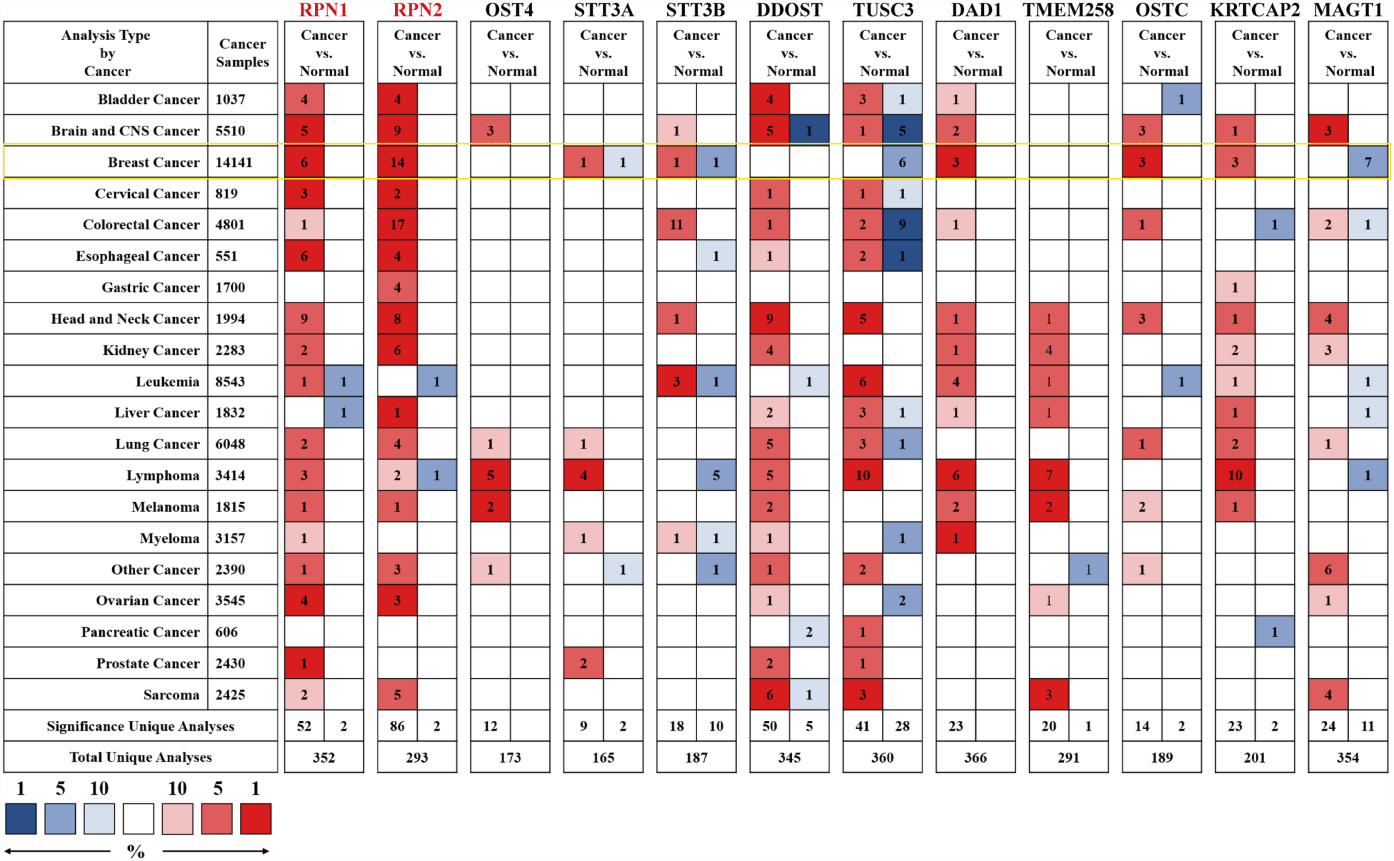
The mRNA expression levels of the OST subunits in different types of human cancers (Oncomine). The figure was generated from Oncomine database with the thresholds that p-value, 0.0001; fold change, 1.5; gene rank: top 10%. The cell number represented the dataset number that met all of the thresholds with the color blue for low expression while the color red for high expression, and the cell color was determined by the best gene rank percentile for the analyses within the cell. An analysis might be counted in more than one cancer type. mRNA expression levels of OST subunits in breast cancer are delineated with yellow highlight. CNS, central nervous system.

We first analyzed the mRNA expression level of OST subunits in different human cancers, especially in BC, compared to the normal breast tissues (**Figure 2**). Analyses that met the threshold were listed in **Supplementary Table S4**. According to **Figure 2** and **Supplementary Table S4**, it could be found that the expression level of RPN1 was upregulated in various subtypes of breast cancer including invasive breast carcinoma, mucinous breast carcinoma, medullary breast carcinoma, invasive ductal breast carcinoma, and ductal breast carcinoma in Curtis’s dataset (22). And the mRNA expression of RPN1 in ductal breast carcinoma was 1.684-fold higher than normal tissues in Sorlie’s dataset (23). The other OST subunit expression patterns were also analyzed in BC tissues. Higher mRNA expression levels of RPN2, DAD1, OSTC, KRTCAP2 and lower expression levels of TUSC3, MAGT1 could be found in different types of BC compared to the normal breast tissues in Curtis’s dataset (22), Zhao’s dataset (24), Ma’s dataset (25), Finak’s dataset (26), Karnoub’s dataset (27), and TCGA dataset.

**Figure 3:**
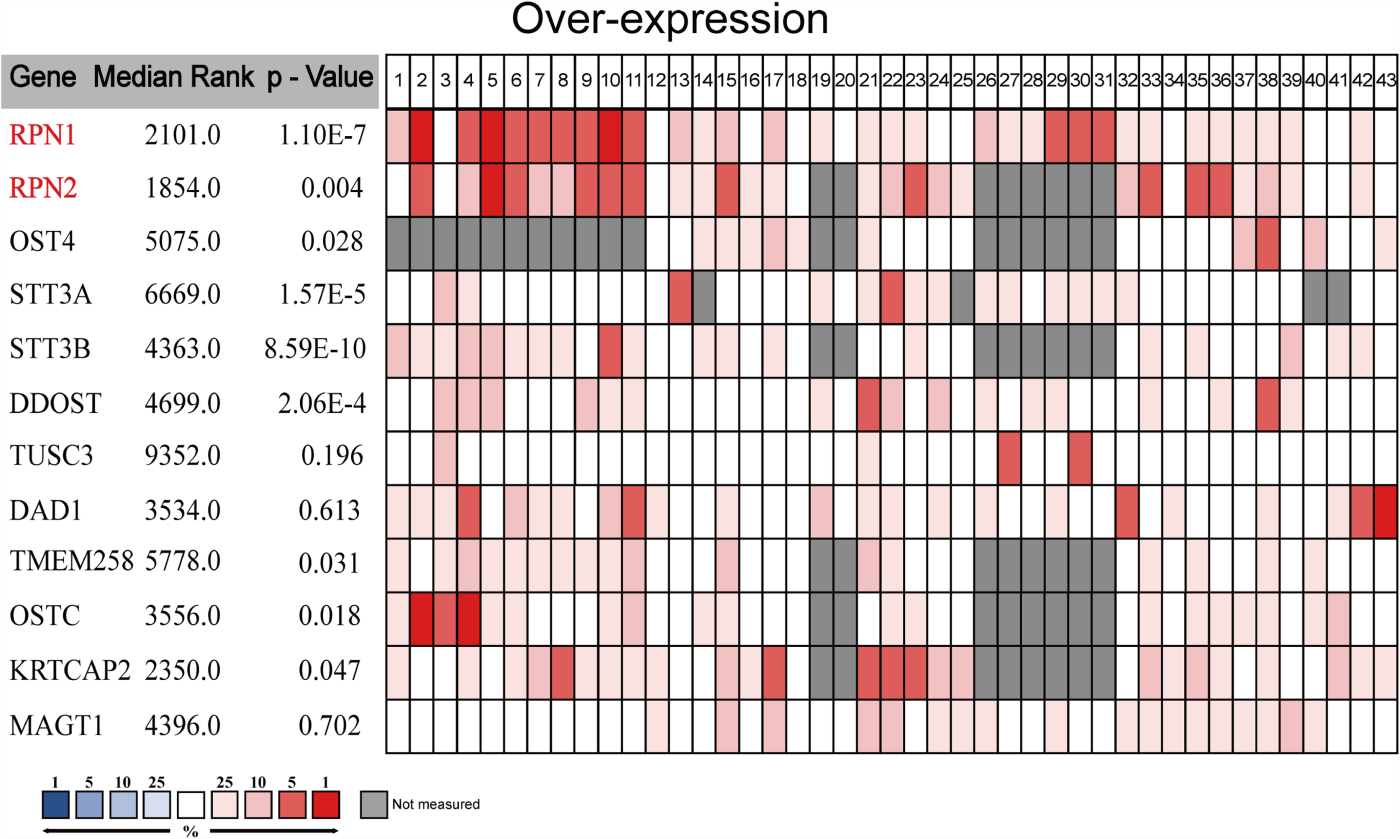
The meta-analysis on the mRNA expression of OST subunits (Oncomine). The comparison of OST subunit mRNA expression in different datasets. Only the upregulated subunits were listed. The rank for a gene was the median rank for that gene across each of the analyses. The p-value for a gene was its p-value for the median-ranked analysis, and p<0.01 was considered statistically significant.

We carried out a meta-analysis by Oncomine and found that only RPN1, RPN2, STT3A, STT3B, and DDOST significantly upregulated in BC tissues according to the 43 analyses of 13 datasets (**Figure 3** and **Supplementary Table S1**).

### The Relationships Between OST Subunits and the Clinicopathologic Features of BC

Then we analyzed the correlations between the mRNA expression of OST subunits especially RPN1, RPN2, STT3A, STT3B, and DDOST, and the clinicopathologic features of BC patients according to Bc-GenExMiner v4.5. The results were presented in **Table 1** and **Supplementary Table S5**. For age character, RPN2 (P = 0.0019) was found to have significantly higher expression in the group not more than 51 years old. For the patient samples with negative estrogen receptor (ER) status, the expression of RPN1, RPN2, STT3A, DDOST were upregulated. The expression levels of RPN1 (P<0.0001), RPN2 (P=0.0003), and DDOST (P<0.0001) were also significantly higher in the BC patient samples with negative progesterone receptor (PR) status. Moreover, compared to the patients with positive human epidermal growth factor receptor 2 (HER2) expression, the mRNA levels of RPN2 (P<0.0001) and STT3B (P<0.0001) were significantly upregulated in the negative ones. In addition, in the BC patients with nodal metastasis, only STT3B (P<0.0001) mRNA expression increased significantly.

**Table 1:** The relationship between the OST subunits and the clinicopathologic parameters of BC (bc-GenExMiner v4.5).

		RPN1		RPN2		STT3A		STT3B		DDOST	
		Comp.	P	Comp.	P	Comp.	P	Comp.	P	Comp.	P
Age	≤51		0.8184	↑	**0.0019**		0.1950		0.0578		0.4482
	>51										
ER (IHC)	Negative	↑	**<0.0001**	↑	**0.0020**	↑	**<0.0001**		0.2391	↑	**<0.0001**
	Positive										
PR (IHC)	Negative	↑	**<0.0001**	↑	**0.0003**		0.1242		0.3620	↑	**<0.0001**
	Positive										
HER2 (IHC)	Negative		0.0661	↑	**<0.0001**		0.1040	↑	**<0.0001**		0.0631
	Positive										
Nodal status	Negative		0.9624		0.1551	↑	**0.0289**		**<0.0001**		0.5209
	Positive							↑			
Intrinsic molecular subtypes	Total		**<0.0001**		**<0.0001**		**<0.0001**		**<0.0001**		**<0.0001**
	Basal-like *vs* Luminal A	>	**<0.0001**	>	**<0.0001**	>	**<0.0001**	>	**<0.0001**	>	**<0.0001**
	Basal-like *vs* Luminal B	>	**<0.0001**	<	**<0.0001**	>	**<0.0001**	<	**<0.0001**	>	**<0.0001**
	Basal-like *vs* HER2-E	>	**<0.05**	<	**<0.0001**	=	>0.01	<	**<0.0001**	>	**<0.0001**
	Luminal B *vs* Luminal A	>	**<0.0001**	>	**<0.0001**	<	**<0.001**	>	**<0.0001**	>	**<0.0001**
	Luminal B *vs* HER2-E	=	>0.01	<	**<0.0001**	<	**<0.0001**	<	**<0.001**	<	**<0.0001**
	HER2-E *vs* Luminal A	>	**<0.0001**	>	**<0.0001**	>	**<0.0001**	>	**<0.0001**	>	**<0.0001**
Basal-like status	Basal	↑	**<0.0001**		0.4492	↑	**<0.0001**		0.2047	↑	**<0.0001**
	None										
SBR	Total		**<0.0001**		**<0.0001**		**0.0012**		**<0.0001**		**<0.0001**
	SBR2 *vs* SBR1	>	**<0.0001**	>	**<0.0001**	=	>0.01	>	**<0.01**	>	**<0.01**
	SBR3 *vs* SBR1	>	**<0.0001**	>	**<0.0001**	>	**<0.01**	>	**<0.0001**	>	**<0.0001**
	SBR3 *vs* SBR2	>	**<0.0001**	>	**<0.0001**	>	**<0.01**	>	**<0.01**	>	**<0.0001**
NPI	Total		**<0.0001**		**0.0015**		0.2014		**0.0024**		0.0715
	NPI2 *vs* NPI1	>	**<0.001**	>	**<0.01**	=	>0.01	>	**<0.01**	=	>0.01
	NPI3 *vs* NPI1	>	**<0.001**	=	>0.01	=	>0.01	=	>0.01	=	>0.01
	NPI3 *vs* NPI2	=	>0.01	=	>0.01	=	>0.01	=	>0.01	=	>0.01

The data with statistical significance (P<0.01) were marked in bold text.

Comp, comparison; IHC, immunohistochemical; ER, estrogen receptor; PR, progesterone receptor; HER2, human epidermal growth factor receptor 2; HER2-E, HER2-enriched; SBR, Scarff Bloom & Richardson grade; NPI, Nottingham Prognostic Index.

Intrinsic molecular subtype is one of the most important clinicopathologic characteristics of BC. The expression levels of all five subunits were significantly higher in basal-like and HER2-enriched patients compared with Luminal A patients, while all five subunits except STT3A expressed higher in Luminal B patients than Luminal A ones. RPN1 and DDOST could be found upregulated in basal-like patients compared with Luminal B and HER2-enriched patients, while RPN2, STT3A, and STT3B were in the opposite. Additionally, it could be found that the expression level of RPN2, STT3A, STT3B, and DDOST increased significantly in Luminal B patients compared with HER2-enriched patients (**Table 1**, **Supplementary Figure S2**).

As the BLBC has the worst prognosis, we especially analyzed the five genes’ mRNA expression between the BLBC and the non-BLBC patients. The expression levels of RPN1 (P<0.0001), STT3A (P<0.0001), and DDOST (P<0.0001) increased significantly in the BLBC compared with non-BLBC patients (**Table 1**, **Supplementary Figure S3**).

In BC, the Scarff Bloom & Richardson (SBR) grade is an important prognostic factor associated with the gland formation, the nuclear features, and the mitotic activity. The SBR is also correlated with poor clinical outcome (28, 29). As shown in **Table 1** and **Supplementary Figure S4**, higher mRNA expression levels of all five genes were associated with a higher SBR grade, while only RPN1, RPN2, STT3B, and DDOST were statistically significant (p<0.01) in all pairwise comparisons. The Nottingham Prognostic Index (NPI) is another system to evaluate the prognosis of BC after surgery, referring to the size of lesion, the number of lymph nodes involved, and the pathologic grade (30, 31). We found that higher expression levels of RPN1, RPN2, and STT3B were associated with higher NPI grade. The expression level of RPN1 was higher in NPI2 and NPI3 patients than in NPI1 patients, but there was no significant difference between NPI2 and NPI3 patients. The expression levels of RPN2 and STT3B increased only in NPI2 patients compared with NPI1 patients (**Supplementary Figure S5**). In summary, the high expression levels of RPN1, RPN2, and STT3B were associated with poor prognosis, suggesting their potential roles in BC.

### Prognostic Values of OST Subunits Expression in BC

**Figure 4:**
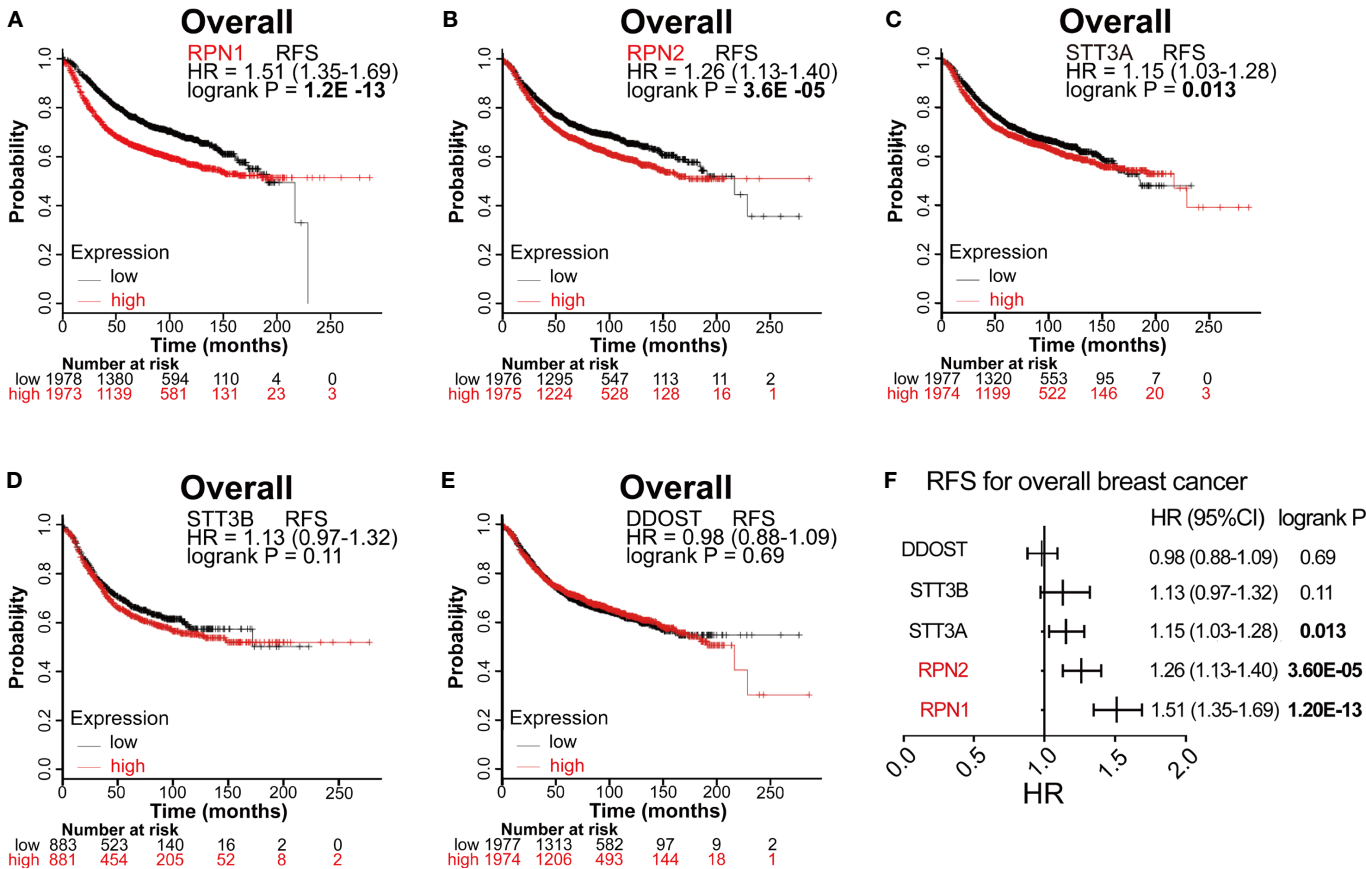
Survival analyses of the five subunits in BC (RFS in Kaplan–Meier Plotter). **(A–E)** RFS for RPN1, RPN2, STT3A, STT3B, and DDOST in all BC. P<0.05 was considered statistically significant. **(F)** Prognostic HR of RFS for the five subunits. The data with statistical significance (P<0.05) were marked in bold text. RFS, relapse-free survival; HR, hazard ratio; CI, confidence interval.

**Figure 5:**
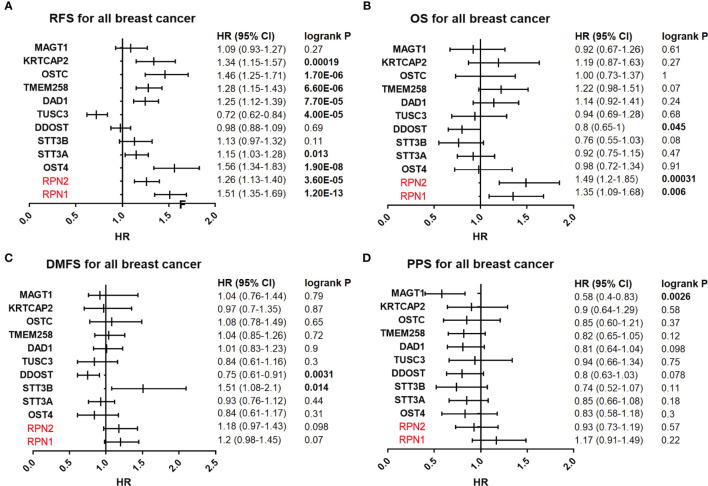
Survival analyses of the OST subunits in breast cancer (RFS, PS, DMFS, PPS in Kaplan–Meier Plotter). **(A–D)** Prognostic HR of RFS, OS, DMFS, and PPS of individual OST subunits in all breast cancers. The data with statistical significance (P<0.05) were marked in bold text. RFS, relapse-free survival; OS, overall survival; DMFS, distant metastasis-free survival; PPS, post-progression survival; HR, hazard ratio; CI, confidence interval.

The prognostic values of all OST subunits in BC were listed in **Supplementary Table S6**, and **Supplementary Figure S6** showed the relapse-free survival (RFS) curves. As for the five selected genes (**Figure 4**), high expression of RPN1 (HR: 1.51, 95% CI: 1.35–1.69, P=1.20E-13), RPN2 (HR: 1.26, 95% CI: 1.13–1.40, P=3.60E-5), and STT3A (HR: 1.15, 95% CI: 1.03–1.28, P=0.013) were associated with worse RFS, while the expression of STT3B (P=0.11) and DDOST (P=0.69) showed no relationship with RFS. We also analyzed the correlation between mRNA expression level of all OST members and other prognostic indexes including overall survival (OS), distant metastasis-free survival (DMFS), and post-progression survival (PPS) (**Supplementary Table S6**, **Figure 5**). High expression level of RPN1 (HR: 1.35, 95% CI: 1.09–1.68, P=0.006) and RPN2 (HR: 1.49, 95% CI: 1.2–1.85, P=0.00031) indicated worse OS, while DDOST (HR: 0.8, 95% CI: 0.65–1.0, P=0045) was in the opposite. High expression level of DDOST (HR: 0.75, 95% CI: 0.61–0.91, P=0.0031) was associated with better DMFS.

We then analyzed the correlation between OST members and prognosis in different subtypes of BC (**Supplementary Table S7** and **Table 2**). In Luminal A patients, high expression of RPN1 (P= 0.00024) and RPN2 (P=9.1E-7) indicated worse RFS. In Luminal B patients, high expression of RPN1 (P=0.025) and STT3A (P=0.028) predicted worse RFS. In HER2-enriched patients, high expression of RPN1(P=0.0093) indicated worse RFS while DDOST (P=0.049) indicated the opposite. In basal-like patients, high expression of RPN1 (P=0.038) and STT3A (P=0.0063) were significantly associated with worse RFS. In a word, these results implied that higher expression of most OST members, especially RPN1 and RPN2, were significantly correlated with poor prognostic outcome and might play a pro-tumor function.

**Table 2:** RFS of the RPN1, RPN2, STT3A, STT3B, and DDOST with different molecular subtypes in breast cancer.

BC subtypes	Gene	Affymetrix ID	Num of patients	HR (95%CI)	logrank P
Basal-like	RPN1	201011_at	618	1.31 (1.01–1.68)	**0.038**
	RPN2	213491_x_at	618	0.87 (0.67–1.12)	0.27
	STT3A	202223_at	618	1.42 (1.1–1.83)	**0.0063**
	STT3B	224700_at	360	1.24 (0.9–1.72)	0.19
	DDOST	208675_s_at	618	0.94 (0.73–1.21)	0.65
Luminal A	RPN1	201011_at	1,933	1.38 (1.16–1.64)	**0.00024**
	RPN2	213491_x_at	1,933	1.54 (1.29–1.83)	**9.1E-07**
	STT3A	202223_at	1,933	0.88 (0.74–1.04)	0.14
	STT3B	224700_at	831	1.12 (0.87–1.43)	0.38
	DDOST	208675_s_at	1,933	0.93 (0.79–1.11)	0.42
Luminal B	RPN1	201011_at	1,149	1.24 (1.03–1.51)	**0.025**
	RPN2	213491_x_at	1,149	1.12 (0.92–1.35)	0.26
	STT3A	202223_at	1,149	1.24 (1.02–1.5)	**0.028**
	STT3B	224700_at	407	1.23 (0.91–1.68)	0.18
	DDOST	208675_s_at	1,149	1 (0.82–1.21)	0.98
HER2-enriched	RPN1	201011_at	251	1.67 (1.13–2.47)	**0.0093**
	RPN2	213491_x_at	251	1.2 (0.82–1.77)	0.35
	STT3A	202223_at	251	1.21 (0.82–1.78)	0.33
	STT3B	224700_at	156	0.75 (0.48–1.18)	0.21
	DDOST	208675_s_at	251	0.68 (0.46–1)	**0.049**

The molecular subtypes were based on the 2013 St Gallen criteria. All of the data above were obtained from the Kaplan-Meier Plotter database.

The data with statistical significance (P<0.05) were marked in bold text.

HR, hazard ratio; CI, confidence interval.

### RPN1 Is a Novel Prognostic Gene for BC

According to the above analyses, it could be concluded that RPN1 and RPN2 were the most influential subunits in BC progression due to their significant relationship between their expression level and clinical prognosis. However, the function of RPN2 in BC has been reported by several studies before (32–34). We focused on the function of RPN1 in BC. Therefore, we analyzed the expression status and prognostic value of RPN1 in BC deeply.

**Figure 6:**
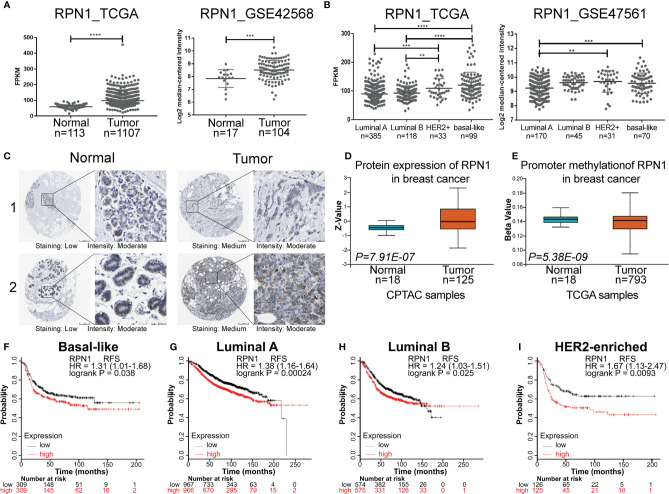
The expression status and prognostic value of RPN1 in BC. **(A)** Gene expression analysis of RPN1 in BC tissues and normal breast tissues according to TCGA database and GEO database (GSE42568). **(B)** Gene expression analysis of RPN1 in different subtypes of BC according to TCGA database and GEO database (GSE47561). **(C)** Representative IHC staining of RPN1 expression in BC tissues and normal tissues according to the HPA database. **(D)** The protein expression of RPN1 in BC tissues and normal tissues in CPTAC samples according to the UALCAN database. Z-values represented standard deviations from the median across samples. **(E)** The promoter methylation level of RPN1 in BC tissues and normal tissues in TCGA samples according to the UALCAN database. The Beta value indicated the level of DNA methylation ranging from 0 (unmethylated) to 1 (fully methylated). **(F–I)** RFS for RPN1 in BLBC, Luminal A, Luminal B, and HER2-enriched BC. P<0.05 was considered statistically significant. **P < 0.01, ***P < 0.001 and ****P < 0.0001.

According to TCGA, GEO (GSE 42568), the HPA, and UALCAN database, both mRNA expression level and protein expression level of RPN1 could be found higher in BC tissues compared to the normal ones (**Figures 6A, C, D**). The details of the IHC figures of RPN1 in the HPA are listed in **Supplementary Table S8**. As mentioned above, BLBC has the worst prognosis. Our analyses showed that the mRNA expression level of RPN1 was the highest in BLBC tissues in TCGA database and the second highest in BLBC tissues in GEO database (GSE47561) (**Figure 6B**). Besides, the promoter methylation level of RPN1 in BC tissues was lower in TCGA samples according to UALCAN database (**Figure 6E**).

In addition, previous result by Kaplan–Meier Plotter analysis has shown that higher mRNA expression of RPN1 indicated worse RFS, and the same tendency could be found in different subtypes of BC (**Figures 6F–I**).

### RPN1 Knockdown Inhibits the Proliferation and Invasion of BLBC Cells

**Figure 7:**
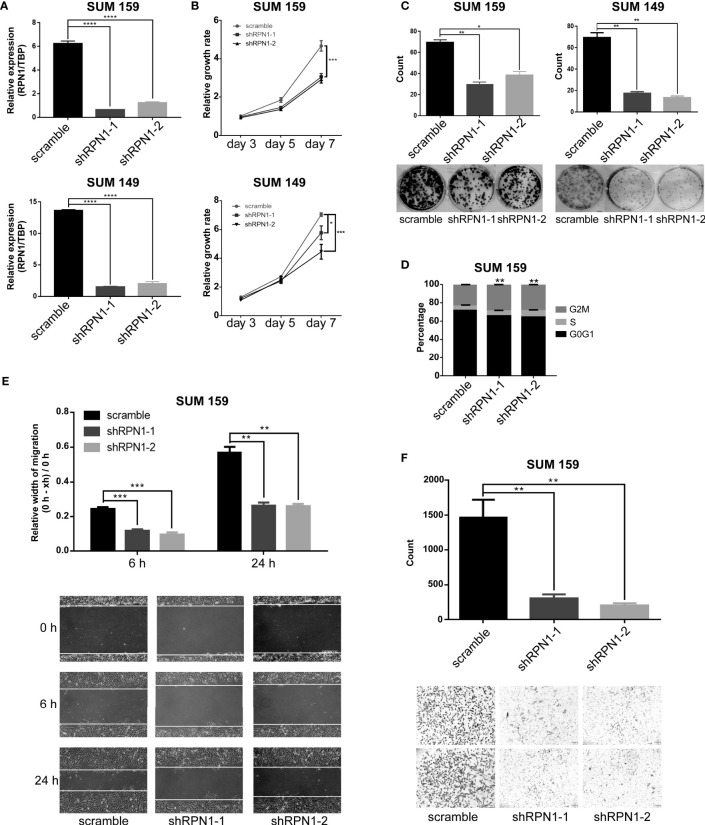
RPN1 knockdown inhibited the growth and invasion of BLBC cells. **(A)** RPN1 was knocked down (scramble was the control). The expression of RPN1 was detected by qRT-PCR in SUM159 and SUM149. **(B, C)** MTT assay and Colony formation assay were used to measure the cell proliferation ability. **(D)** Cell cycle distribution analyzed by flow cytometry in SUM159 cells. **(E, F)** Wound healing assay and transwell assay were used to measure the cell migration and invasion ability. *P < 0.05, **P < 0.01, ***P < 0.001 and ****P < 0.0001.

To explore the function of RPN1 in BLBC, we established shRNA-mediated RPN1 knockdown cell lines in SUM159 and SUM149, the two BLBC cell lines (**Figure 7A**). RPN1 knockdown induced significant proliferation inhibition (**Figures 7B, C**), which might be due to the cell cycle arrest, because of the remarkably increased percentage of cells in G2/M phase (**Figure 7D**). In addition, migration and invasion abilities of SUM159 cells were significantly inhibited by the knockdown of RPN1 (**Figures 7E, F**).

### RPN1 Knockdown Induces ERS-Dependent Cell apoptosis in BLBC

**Figure 8:**
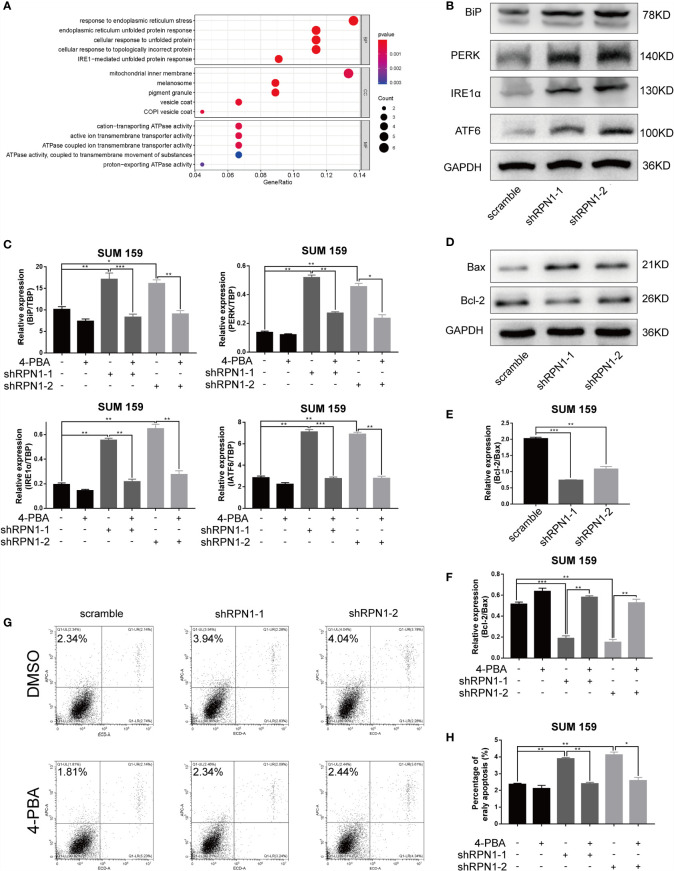
ERS-dependent apoptosis was triggered by the knockdown of RPN1. **(A)** Bubble plot of the GO function enrichment analysis of the genes positively correlated with RPN1 RNA expression level in BLBC. Y-axis represents the name of the function, and X-axis represents the ratio of the number of the genes assigned to a term to the total number of the genes. The Bubble size represents the number of the genes annotated to the function. The color of the bubble represents the enriched P-value, while the red indicates a greater significance level. **(B)** The expression of ERS-related proteins was detected by western blot in SUM159 cells. **(C)** Scramble and shRPN1-infuected SUM159 cells were treated with 4-PBA (2 uM) or same volume of DMSO for 48 h, and the ERS markers were determined by qRT-PCR. **(D, E)** The protein expression of Bax and Bcl-2 in SUM159 detected by western blot and the ratio of the protein expression of Bcl-2 and Bax were also shown. **(F)** The ratio of the mRNA expression level of Bcl-2 and Bax was determined by qRT-PCR in 4-PBA- or DMSO-treated SUM159 cells. **(G, H)** Apoptosis analyzed by flow cytometry in 4-PBA- or DMSO-treated SUM159 cells. *P < 0.05, **P < 0.01 and ***P < 0.001. BP, Biological process; CC, cellular component; MF, molecular function; 4-PBA, Sodium phenylbutyrate.

To explore the possible mechanisms of RPN1 in regulating the proliferation and invasion of BC cells, a total of 46 positively co-expressed genes of RPN1 with a Pearson correlation no less than 0.40 were obtained from the RNA-seq data in BLBC by bc-GenExMiner v4.5 database (**Supplementary Table S9**). And the GO enrichment analyses revealed that the biological process of “response to ERS”, “endoplasmic reticulum unfolded protein response”, “cellular response to unfolded protein”, “cellular response to topologically incorrect protein”, and “IRE1α-mediated unfolded protein response” were enriched for these genes (**Figure 8A**), indicating the possible important role of ERS in the knockdown of RPN1.

RPN1 plays a critical role in N-linked glycosylation, and previous studies have shown that the abnormality of the N-linked glycosylation may induce ERS in cells. Though the effect of ERS on tumor growth and metastasis was complex and dynamic, it has been proven that ERS could inhibit the growth and metastasis of tumors (35, 36). Inositol-requiring protein 1 α (IRE1α), protein kinase RNA -like endoplasmic reticulum kinase (PERK), and activating transcription factor 6 (ATF6) are endoplasmic reticulum transmembrane proteins, and each of them mediates an arm of the UPR. Normally, they are in a silent state combining with the endoplasmic reticulum chaperone immunoglobulin-binding protein (BiP). When under the ERS, they dissociate from BiP and activate their signaling functions respectively (14, 37). The results of both western blot and qRT-PCR showed the upregulation of PERK, IRE1α, ATF6, and BiP in RPN1-knockdown SUM159 cells (**Figures 8B, C**), suggesting that the ERS was induced after knockdown of RPN1. The ERS inhibitor 4-PBA could interact with unfolded or misfolded proteins to alleviate ERS (38). Treated with 4-PBA, the ERS could be significantly reduced in RPN1-knockdown SUM159 cells (**Figure 8C**). Several studies have demonstrated that the persistent ERS could play a pro-death role and trigger apoptosis (14, 35). Here, we found the knockdown of RPN1 decreased Bcl-2/Bax ratio at both protein and mRNA levels, which meant an increased apoptosis (**Figures 8D–F**), while treatment of 4-PBA increased the Bcl-2/Bax ratio (**Figure 8F**). We also found that the knockdown of RPN1 induced significant increase of early apoptosis in SUM159 cells, while treatment of 4-PBA rescued it (**Figures 8G, H**). These results demonstrated that inhibition of RPN1 could suppress BLBC cell proliferation and invasion *via* triggering the ERS.

## Discussion

N-glycosylation, one of important ways of post-translational modification, plays an important role in maintaining the stability of proteins. Most secreted proteins require glycosylation to maintain stability and solubility, and N-glycosylation could assist proteins forming a proper folded structure by increasing the hydrophilicity of them or determining the chaperone bound to them (39). The OST complex is important for N-glycosylation, the abnormality of which is involved in tumors. Liu et al. found that the N-glycan profiles of membrane proteins in BC tissues significantly changed compared to the adjacent normal ones (40). Furthermore, previous reports have demonstrated the N-glycan alterations were essential for tumorigenesis, proliferation, and metastasis *via* modifying critical proteins or triggering mechanisms involved in the maintenance of cell homeostasis, such as ERS (41–45).

The 12 known subunits of OST complex play different roles in N-glycosylation. Some of the subunits have been reported to be associated with tumor. Takahashi et al. found that RPN2 could stabilize mutant p53 by inactivation of glycogen synthase kinase-3b, and the overexpression of RPN2 promoted the growth of BC (32). Burgermeister et al. revealed that the silence of TUSC3 by methylation was associated with the tumorigenesis of colorectal cancer, and epidermal growth factor receptor could be one of the target proteins (46).

In our study, we found that the mRNA expression levels of RPN1, RPN2, STT3A STT3B, and DDOST were significantly upregulated in BC tissues, and the expression levels of RPN1 and DDOST were significantly higher in the BLBC tissues compared to the non-BLBC. As for SBR and NPI, with the increasing of the grade of both SBR and NPI, the expression levels of RPN1, RPN2, and STT3B increased. As for the survival, the high expression of RPN1, RPN2, and STT3A were associated with worse RFS. Considering about both expression level and survival value, RPN1 and RPN2 could be the most effective biomarker and the most potential therapeutic target of OST subunits in BC. However, some studies have revealed that RPN2 plays a critical role in different cancers (32–34, 47, 48), while there was almost no study reporting the effect of RPN1.

RPN1 has been confirmed to be a type I transmembrane protein located on the endoplasmic reticulum, regulating N-glycosylation by interaction with the ribosomes and facilitating the specific precursors to the catalytic STT3A and STT3B subunits as a chaperone (15, 49). We conducted *in-vitro* experiments after knockdown of RPN1 in cells. And it turned out that the knockdown of RPN1 by shRNA led to poorer proliferation rate and less migration as well as invasion.

ERS is a mechanism to maintain the homeostasis of cell. And the aberrant glycosylation of proteins can lead to ERS and activate a set of signaling pathways (6). As mentioned previously, PERK, IRE1α, and ATF6 mediate three arms of UPR independently, and the signal pathways initiated by them could induce cell apoptosis. PERK, as a Ser/Thr kinase, mediates phosphorylation of eukaryotic initiation factor 2 (eIF2α) and then leads to the translation of transcription factor ATF4 (50). IRE1α can act not only as a protein kinase but also as an endoribonuclease. On the one hand, IRE1α can activate a pathway leading to c-Jun N-terminal kinase phosphorylation, which can promote apoptosis in several pathways (51). On the other hand, IRE1α is able to splice the mRNA of the transcription factor X-Box Binding Protein 1 (XBP1), producing XBP1s (52). ATF6 could not only cleave itself as a downstream signal molecule but also induce the modification of XBP1 (53). C/EBP homologous protein (CHOP), as an important pro-apoptotic transcription factor, can be the shared target of the three branches of UPR. It can be upregulated by the increased of ATF4, XBP1s, and cleaved ATF6. CHOP could induce the upregulation of various essential genes including Bcl-2 family members (54), thereby increase cell apoptosis directly.

The role ERS plays in tumorigenesis, proliferation, invasion, and apoptosis has been extensively reported (35, 55). In our study, we found that the knockdown of RPN1 inhibited the proliferation, migration, and invasion of BC. And the knockdown of RPN1 induced the upregulation of BiP, PERK, IRE1α, and ATF6 and the increase of cell apoptosis, while the treatment of ERS inhibitor could rescue them. These phenomena indicated that the RPN1 played a pro-tumor role by maintaining the endoplasmic reticulum homoeostasis in BLBC cells. However, the main target of RPN1 and the specific downstream pathway of ERS need further exploration.

In conclusion, clinically, the high expression level of RPN1 not only predicts a worse prognosis but is also related to a variety of recognized indicators of poor prognosis like negative ER status, negative PR status, BLBC subtype, higher SBR, and higher NPI. Biologically, our *in vitro* experiments clearly confirm that the proliferation, migration, and invasion of BC cells are significantly inhibited after interfering the expression of RPN1. Mechanismly, RPN1 inhibition leads to the activation of ERS and subsequent cell apoptosis. Although the detailed molecular mechanism is still not clear, it can be apparent that RPN1 plays an important part in BC and may be a novel biomarker as well as a potential therapeutic target.

## Data Availability Statement

The original contributions presented in the study are included in the article/**Supplementary Material**. Further inquiries can be directed to the corresponding authors.

## Author Contributions

JD analyzed the databases and focused this study on RPN1 and was the major contributor in writing the manuscript. JX finished the most *in-vitro* experiments and contributed a lot in the manuscript. QD and XH contributed a lot in the experiments. RZ verified the accuracy of all the results and collated all the pictures. LX and SL were responsible for final approval of the version to be submitted and are accountable for all aspects of the work in ensuring that questions related to the accuracy or integrity of any part of the manuscript are appropriately investigated and resolved. And all authors commented on previous versions of the manuscript. All authors contributed to the article and approved the submitted version.

## Funding

The work was supported by funds from the National Key Research and Development Program of China (2018YFA0507501, 2020YFA0112300), NSFC Grant (81530075, 82073067, 81773155, 81772799), Program for Outstanding Medical Academic Leader 2019LJ04, Fudan University Research Foundation (IDH1340042), and Research Foundation of the Fudan University Shanghai Cancer Center (YJRC1603).

## Conflict of Interest

The authors declare that the research was conducted in the absence of any commercial or financial relationships that could be construed as a potential conflict of interest.

## Publisher’s Note

All claims expressed in this article are solely those of the authors and do not necessarily represent those of their affiliated organizations, or those of the publisher, the editors and the reviewers. Any product that may be evaluated in this article, or claim that may be made by its manufacturer, is not guaranteed or endorsed by the publisher.

## Supplementary Material

Supplementary Figure S1 Protein-protein interaction network of OST family members (data from GeneMANIA). Protein-protein interaction network among OST subunits. Each node indicates a gene, and the node size represents the strength of interactions. The internode connection lines represent the types of gene-gene interactions, and the line color represents the types of interactions, while the color of node represents the possible functions of these genes.

Supplementary Figure S2 The relationships between the OST subunits and intrinsic molecular subtypes of breast cancer (data from bc-GenExMiner v4.7). **(A–L)** Box plots of individual OST subunit’s expression according to the intrinsic molecular subtype of breast cancer (including basal-like, luminal A, luminal B, and HER2-enriched). Significant differences between groups were assessed by Welch’s test, and Dunnett–Tukey–Kramer’s test computed for each pairwise comparison. P<0.05 was considered statistically significant. In addition, the data in this figure was obtained from bc-GenExMiner v4.7 due to the update of the website. HER2-E, human epidermal growth factor receptor 2 enriched.

Supplementary Figure S3 The relationships between the OST subunits and basal-like status of breast cancer (data from bc-GenExMiner v4.5). **(A–L)** Box plots of individual OST subunit’s expression according to the basal-like status of breast cancer (basal-like or not). Significant differences between groups were assessed by Welch’s test, and P<0.05 was considered statistically significant.

Supplementary Figure S4 The relationships between the OST subunits and the SBR criteria (data from bc-GenExMiner v4.5). **(A–L)** Box plots of individual OST subunit’s expression according to SBR. Global significant differences between groups were assessed by Welch’s test, and Dunnett–Tukey–Kramer’s test computed for each pairwise comparison. P<0.05 was considered statistically significant. SBR, Scarff Bloom & Richardson grade.

Supplementary Figure S5 The relationships between the OST subunits and the NPI criteria (data from bc-GenExMiner v4.5). **(A–L)** Box plots of individual OST subunit’s expression according to NPI. Global significant differences between groups were assessed by Welch’s test, and Dunnett–Tukey–Kramer’s test computed for each pairwise comparison. P<0.05 was considered statistically significant. NPI, Nottingham Prognostic Index.

Supplementary Figure S6 Survival analyses of the OST subunits in breast cancer (RFS in Kaplan–Meier Plotter). **(A–L)** RFS for individual OST subunits in all breast cancers. P<0.05 was considered statistically significant. RFS, relapse-free survival; HR, hazard ratio.
