# Supplementary figures and images for: Knockdown of Oligosaccharyltransferase Subunit Ribophorin 1 Induces Endoplasmic-Reticulum-Stress-Dependent Cell Apoptosis in Breast Cancer
Source: Front Oncol. 2021 Oct 27;11:722624. doi: 10.3389/fonc.2021.722624 (PMC8578895; doi:10.3389/fonc.2021.722624)

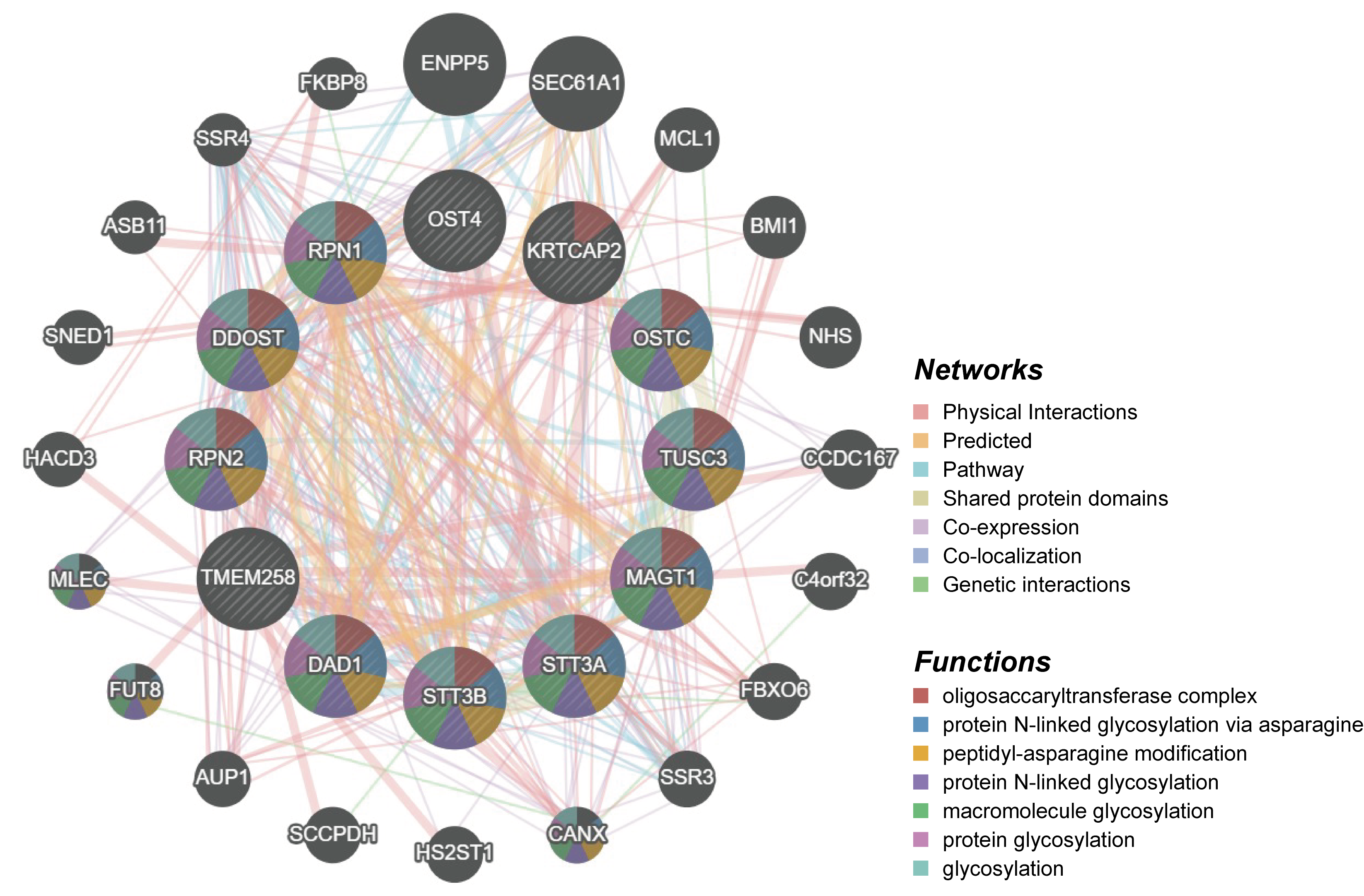

Supplement: Supplementary Figure S1 — Protein-protein interaction network of OST family members (data from GeneMANIA). Protein-protein interaction network among OST subunits. Each node indicates a gene, and the node size represents the strength of interactions. The internode connection lines represent the types of gene-gene interactions, and the line color represents the types of interactions, while the color of node represents the possible functions of these genes. [file Image_1.tif]

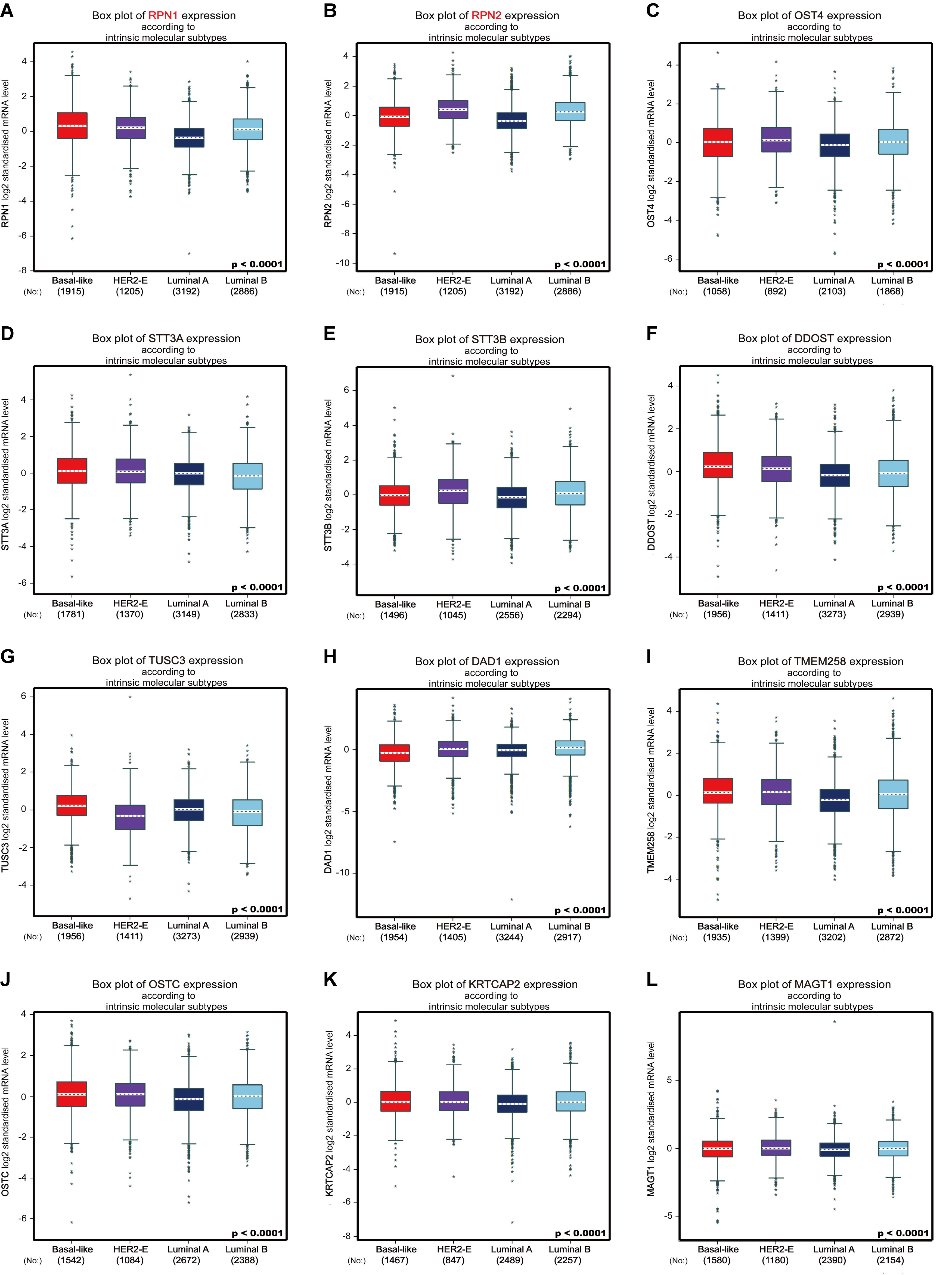

Supplement: Supplementary Figure S2 — The relationships between the OST subunits and intrinsic molecular subtypes of breast cancer (data from bc-GenExMiner v4.7). (A–L) Box plots of individual OST subunit’s expression according to the intrinsic molecular subtype of breast cancer (including basal-like, luminal A, luminal B, and HER2-enriched). Significant differences between groups were assessed by Welch’s test, and Dunnett–Tukey–Kramer’s test computed for each pairwise comparison. P<0.05 was considered statistically significant. In addition, the data in this figure was obtained from bc-GenExMiner v4.7 due to the update of the website. HER2-E, human epidermal growth factor receptor 2 enriched. [file Image_2.tif]

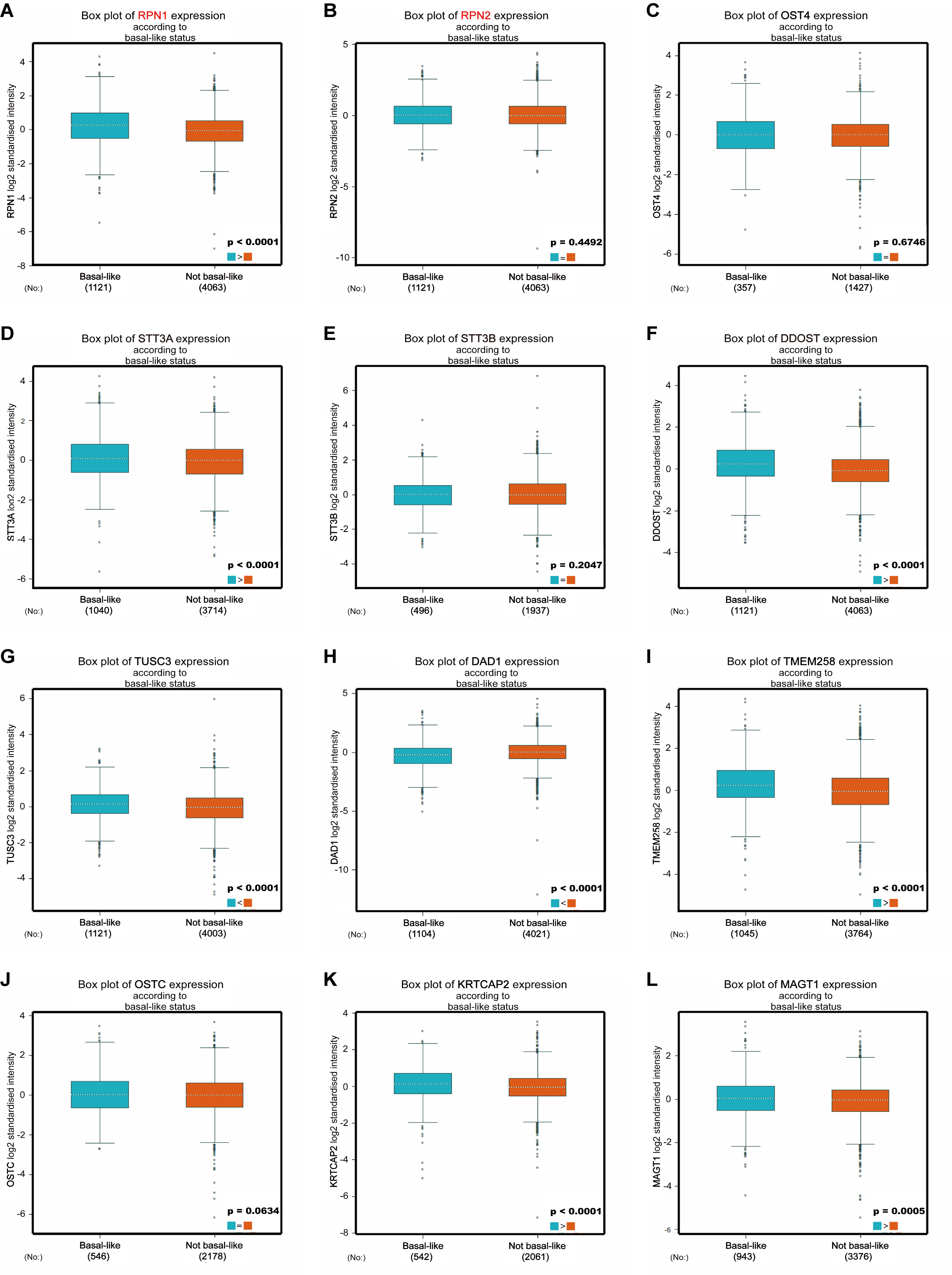

Supplement: Supplementary Figure S3 — The relationships between the OST subunits and basal-like status of breast cancer (data from bc-GenExMiner v4.5). (A–L) Box plots of individual OST subunit’s expression according to the basal-like status of breast cancer (basal-like or not). Significant differences between groups were assessed by Welch’s test, and P<0.05 was considered statistically significant. [file Image_3.tif]

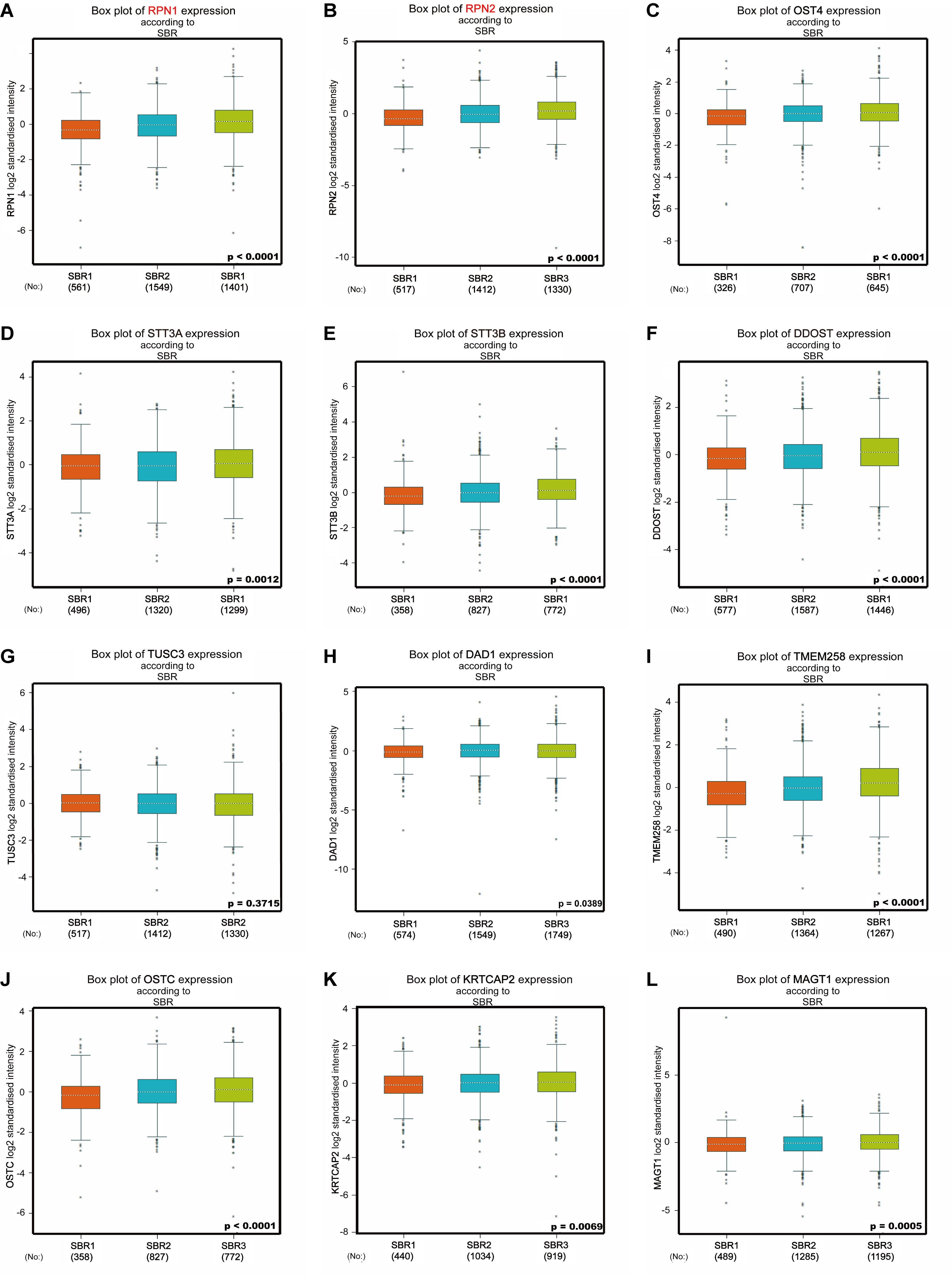

Supplement: Supplementary Figure S4 — The relationships between the OST subunits and the SBR criteria (data from bc-GenExMiner v4.5). (A–L) Box plots of individual OST subunit’s expression according to SBR. Global significant differences between groups were assessed by Welch’s test, and Dunnett–Tukey–Kramer’s test computed for each pairwise comparison. P<0.05 was considered statistically significant. SBR, Scarff Bloom & Richardson grade. [file Image_4.tif]

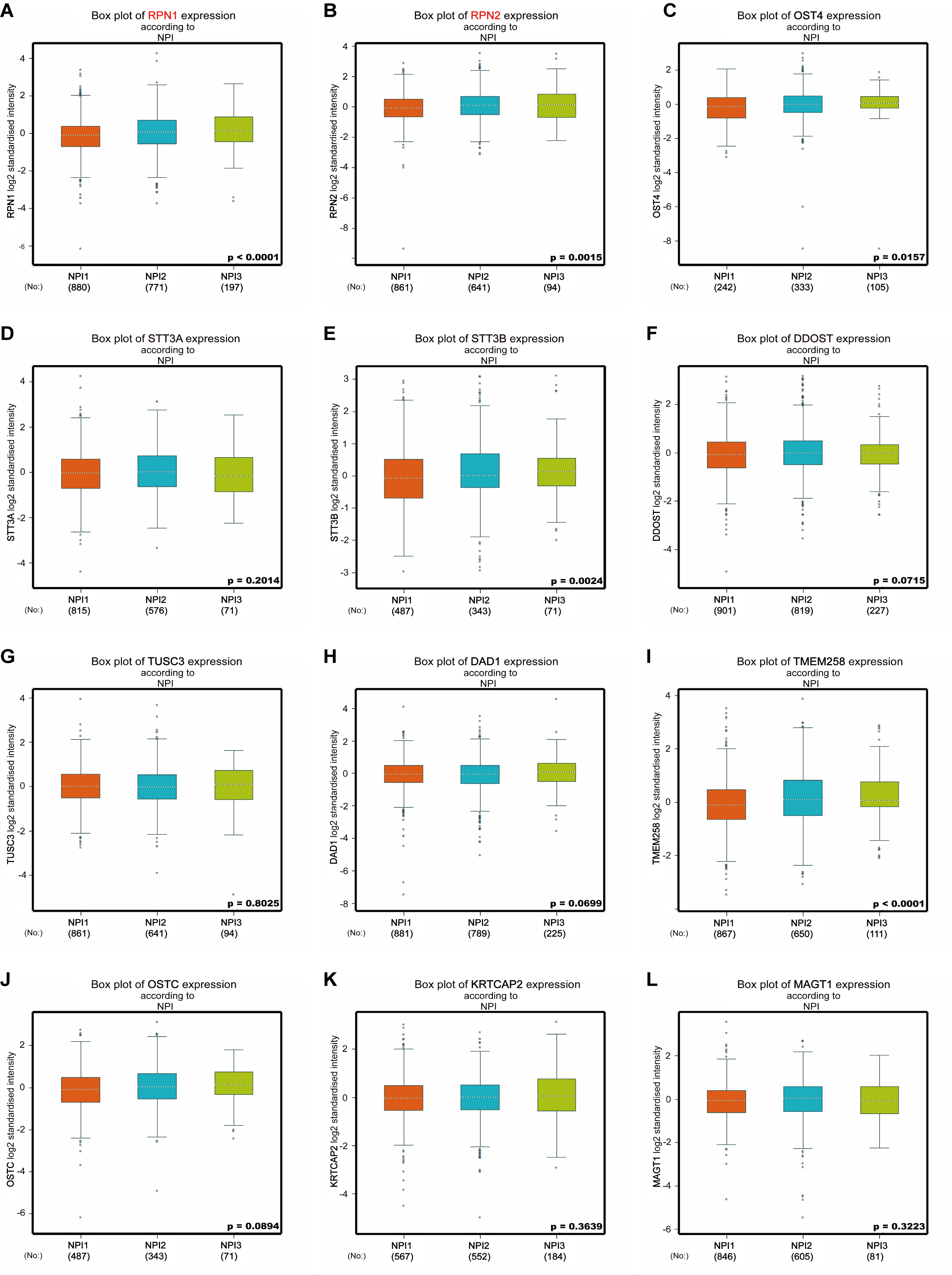

Supplement: Supplementary Figure S5 — The relationships between the OST subunits and the NPI criteria (data from bc-GenExMiner v4.5). (A–L) Box plots of individual OST subunit’s expression according to NPI. Global significant differences between groups were assessed by Welch’s test, and Dunnett–Tukey–Kramer’s test computed for each pairwise comparison. P<0.05 was considered statistically significant. NPI, Nottingham Prognostic Index. [file Image_5.tif]

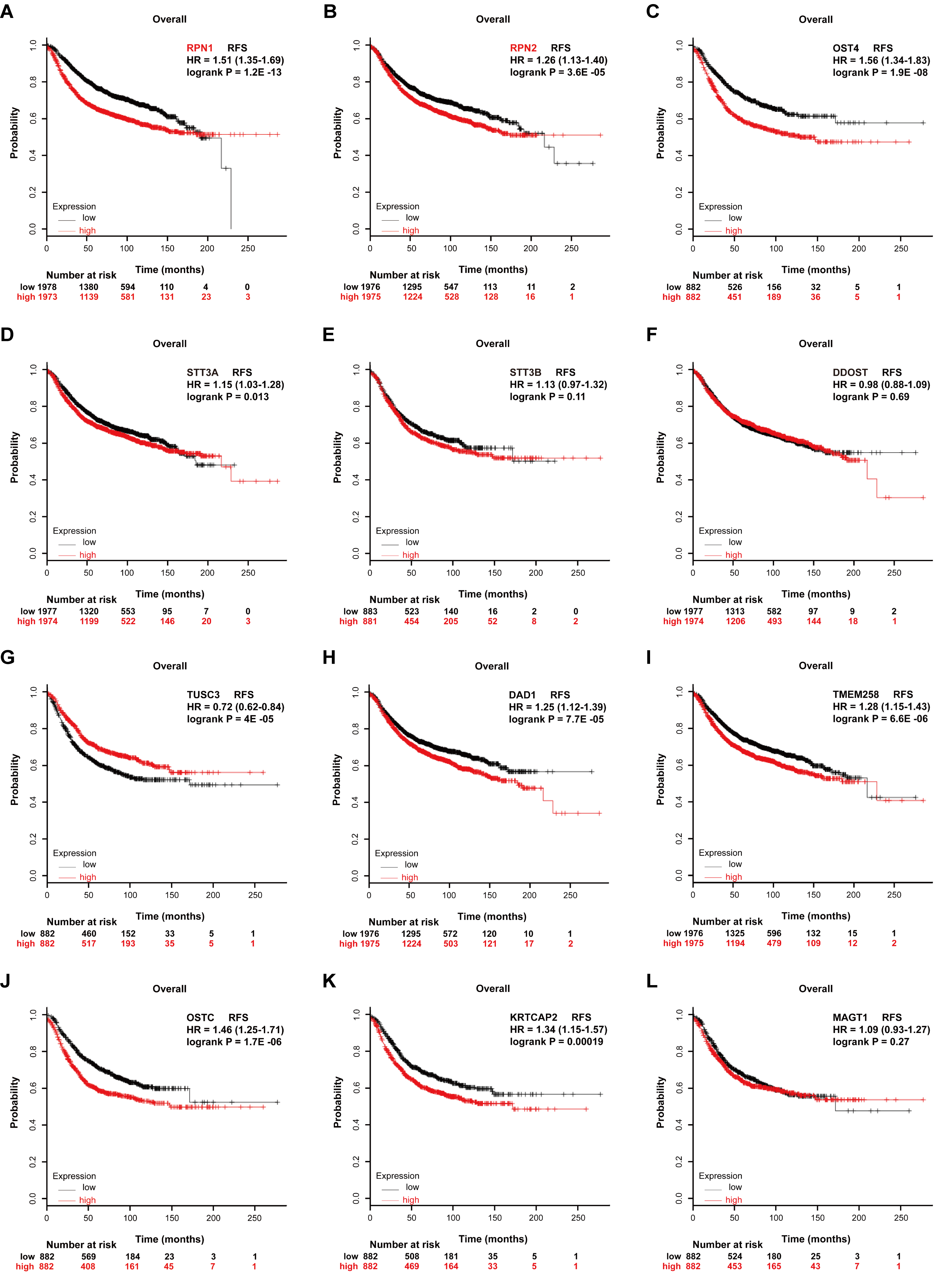

Supplement: Supplementary Figure S6 — Survival analyses of the OST subunits in breast cancer (RFS in Kaplan–Meier Plotter). (A–L) RFS for individual OST subunits in all breast cancers. P<0.05 was considered statistically significant. RFS, relapse-free survival; HR, hazard ratio. [file Image_6.tif]
