# Supplementary material for: Knockdown of Oligosaccharyltransferase Subunit Ribophorin 1 Induces Endoplasmic-Reticulum-Stress-Dependent Cell Apoptosis in Breast Cancer
Source: Front Oncol. 2021 Oct 27;11:722624. doi: 10.3389/fonc.2021.722624 (PMC8578895; doi:10.3389/fonc.2021.722624)
Supplement: Supplementary file 7 [file Table_1.docx]

**Table S1** The details of the 43 analyzes of 13 datasets on BC (Oncomine database)

| Number | Dataset | Analysis | Associated Paper |
| --- | --- | --- | --- |
| 1 | Curtis Breast | Benign Breast Neoplasm (3) vs. Normal (144) | Nature, 2012 |
| 2 |  | Breast Carcinoma (14) vs. Normal (144) |  |
| 3 |  | Breast Phyllodes Tumor (5) vs. Normal (144) |  |
| 4 |  | Ductal Breast Carcinoma in Situ (10) vs. Normal (144) |  |
| 5 |  | Invasive Breast Carcinoma (21) vs. Normal (144) |  |
| 6 |  | Invasive Ductal and Invasive Lobular Breast Carcinoma (90) vs. Normal (144) |  |
| 7 |  | Invasive Ductal Breast Carcinoma (1556) vs. Normal (144) |  |
| 8 |  | Invasive Lobular Breast Carcinoma (148) vs. Normal (144) |  |
| 9 |  | Medullary Breast Carcinoma (32) vs. Normal (144) |  |
| 10 |  | Mucinous Breast Carcinoma (46) vs. Normal (144) |  |
| 11 |  | Tubular Breast Carcinoma (67) vs. Normal (144) |  |
| 12 | Finak Breast | Invasive Breast Carcinoma Stroma (53) vs. Normal (6) | Nat Med, 2008 |
| 13 | Gluck Breast | Invasive Breast Carcinoma (154) vs. Normal (4) | Breast Cancer Res Treat, 2011 |
| 14 | Karnoub Breast | Invasive Ductal Breast Carcinoma Stroma (7) vs. Normal (15) | Nature, 2007 |
| 15 | Ma Breast 4 | Ductal Breast Carcinoma in Situ Epithelia (20) vs. Normal (28) | Breast Cancer Res, 2009 |
| 16 |  | Ductal Breast Carcinoma in Situ Stroma (20) vs. Normal (28) |  |
| 17 |  | Invasive Ductal Breast Carcinoma Epithelia (18) vs. Normal (28) |  |
| 18 |  | Invasive Ductal Breast Carcinoma Stroma (18) vs. Normal (28) |  |
| 19 | Perou Breast | Ductal Breast Carcinoma (55) vs. Normal (3) | Nature, 2000 |
| 20 |  | Lobular Breast Carcinoma (4) vs. Normal (3) |  |
| 21 | Radvanyi Breast | Ductal Breast Carcinoma in Situ (3) vs. Normal (7) | Proc Natl Acad Sci U S A, 2005 |
| 22 |  | Invasive Ductal Breast Carcinoma (31) vs. Normal (7) |  |
| 23 |  | Invasive Lobular Breast Carcinoma (7) vs. Normal (7) |  |
| 24 |  | Invasive Mixed Breast Carcinoma (3) vs. Normal (7) |  |
| 25 | Richardson Breast 2 | Ductal Breast Carcinoma (40) vs. Normal (7) | Cancer Cell, 2006 |
| 26 | Sorlie Breast | Ductal Breast Carcinoma (68) vs. Normal (4) | Proc Natl Acad Sci U S A, 2001 |
| 27 |  | Fibroadenoma (3) vs. Normal (4) |  |
| 28 |  | Lobular Breast Carcinoma (5) vs. Normal (4) |  |
| 29 | Sorlie Breast 2 | Ductal Breast Carcinoma (140) vs. Normal (4) | Proc Natl Acad Sci U S A, 2003 |
| 30 |  | Fibroadenoma (3) vs. Normal (4) |  |
| 31 |  | Lobular Breast Carcinoma (11) vs. Normal (4) |  |
| 32 | TCGA Breast | Intraductal Cribriform Breast Adenocarcinoma (3) vs. Normal (61) | No Associated Paper, 2011 |
| 33 |  | Invasive Breast Carcinoma (76) vs. Normal (61) |  |
| 34 |  | Invasive Ductal and Lobular Carcinoma (3) vs. Normal (61) |  |
| 35 |  | Invasive Ductal Breast Carcinoma (392) vs. Normal (61) |  |
| 36 |  | Invasive Lobular Breast Carcinoma (36) vs. Normal (61) |  |
| 37 |  | Male Breast Carcinoma (3) vs. Normal (61) |  |
| 38 |  | Mixed Lobular and Ductal Breast Carcinoma (7) vs. Normal (61) |  |
| 39 |  | Mucinous Breast Carcinoma (4) vs. Normal (61) |  |
| 40 | Turashvili Breast | Invasive Ductal Breast Carcinoma (5) vs. Normal (20) | BMC Cancer, 2007 |
| 41 |  | Invasive Lobular Breast Carcinoma (5) vs. Normal (20) |  |
| 42 | Zhao Breast | Invasive Ductal Breast Carcinoma (40) vs. Normal (3) | Mol Biol Cell, 2004 |
| 43 |  | Lobular Breast Carcinoma (21) vs. Normal (3) |  |

**Note:** The number of samples in each analysis was listed in the parenthese
