# Supplementary material for: Knockdown of Oligosaccharyltransferase Subunit Ribophorin 1 Induces Endoplasmic-Reticulum-Stress-Dependent Cell Apoptosis in Breast Cancer
Source: Front Oncol. 2021 Oct 27;11:722624. doi: 10.3389/fonc.2021.722624 (PMC8578895; doi:10.3389/fonc.2021.722624)
Supplement: Supplementary file 8 [file Table_2.docx]

**Table S2** PLKO.1 ShRNA sequences

| shRPN1-1 | Forward | ATCTCGAGATTTCGCTCACTCTGTCGCACTTTTTTGAATTCTCGACCTCGAG |
| --- | --- | --- |
|  | Reverse | AAATCTCGAGATTTCGCTCACTCTGTCGCACCGGTGTTTCGTCCTTTCC |
| shRPN1-2 | Forward | TACTCGAGTAATCATAGCGTGAGAAAGGCTTTTTTGAATTCTCGACCTCGAG |
|  | Reverse | ATTACTCGAGTAATCATAGCGTGAGAAAGGCCGGTGTTTCGTCCTTTCC |
