# Supplementary material for: Knockdown of Oligosaccharyltransferase Subunit Ribophorin 1 Induces Endoplasmic-Reticulum-Stress-Dependent Cell Apoptosis in Breast Cancer
Source: Front Oncol. 2021 Oct 27;11:722624. doi: 10.3389/fonc.2021.722624 (PMC8578895; doi:10.3389/fonc.2021.722624)
Supplement: Supplementary file 9 [file Table_3.docx]

**Table S3** The primers for qRT-PCR

| RPN1 | Forward | GGCCTTTCAGAGATGTGCCT |
| --- | --- | --- |
|  | Reverse | GCAGCAGGAAGGATGGTCTT |
| ATF6 | Forward | CTTTTAGCCCGGGACTCTTT |
|  | Reverse | TCAGCAAAGAGAGCAGAATCC |
| IRE1α | Forward | GAAGCATGTGCTCAAACACC |
|  | Reverse | TCTGTCGCTCACGTCCTG |
| PERK | Forward | CCAGCCTTAGCAAACCAGAG |
|  | Reverse | TCTTGGTCCCACTGGAAGAG |
| BiP | Forward | CATCAAGTTCTTGCCGTTCA |
|  | Reverse | TCTTCAGGAGCAAATGTCTTTGT |
| Bax | Forward | CCCGAGAGGTCTTTTTCCGAG |
|  | Reverse | CCAGCCCATGATGGTTCTGAT |
| Bcl-2 | Forward | ACAGGGTACGATAACCGGGA |
|  | Reverse | GCCCAGACTCACATCACCAA |
| TBP | Forward | TGCACAGGAGCCAAGAGTGAA |
|  | Reverse | CACATCACAGCTCCCCACCA |
