# Supplementary material for: Knockdown of Oligosaccharyltransferase Subunit Ribophorin 1 Induces Endoplasmic-Reticulum-Stress-Dependent Cell Apoptosis in Breast Cancer
Source: Front Oncol. 2021 Oct 27;11:722624. doi: 10.3389/fonc.2021.722624 (PMC8578895; doi:10.3389/fonc.2021.722624)
Supplement: Supplementary file 10 [file Table_4.docx]

**Table S4** Datasets of the OST subunits in breast cancer (oncomine database)

| **Gene** | **Dataset** | **Normal（cases）** | **Tumor（cases）** | **Fold change** | **t-test** | **P-value** | **Gene Rank** |
| --- | --- | --- | --- | --- | --- | --- | --- |
| **RPN1** | Curtis Breast | Breast (144) | Invasive Breast Carcinoma (21) | **1.577** | 10.734 | **2.22E-11** | 7 |
|  |  | Breast (144) | Mucinous Breast Carcinoma (46) | **1.561** | 14.741 | **1.76E-24** | 34 |
|  |  | Breast (144) | Medullary Breast Carcinoma (32) | **1.833** | 11.300 | **8.81E-14** | 235 |
|  |  | Breast (144) | Invasive Ductal Breast Carcinoma (1,556) | **1.590** | 28.293 | **1.17E-71** | 597 |
|  |  | Breast (144) | Ductal Breast Carcinoma in Situ (10) | **1.510** | 5.086 | **2.64E-4** | 742 |
|  | Sorlie Breast 2 | Breast (4) | Ductal Breast Carcinoma (94) | **1.684** | 6.802 | **8.66E-4** | 296 |
| **RPN2** | Curtis Breast | Breast (144) | Invasive Breast Carcinoma (21) | **2.589** | 7.766 | **3.57E-8** | 151 |
|  |  | Breast (144) | Mucinous Breast Carcinoma (46) | **2.439** | 11.486 | **1.62E-17** | 310 |
|  |  | Breast (144) | Breast Carcinoma (14) | **1.792** | 6.073 | **7.28E-6** | 457 |
|  |  | Breast (144) | Medullary Breast Carcinoma (32) | **2.355** | 8.631 | **7.89E-11** | 660 |
|  |  | Breast (144) | Invasive Ductal and Invasive Lobular Breast Carcinoma (90) | **1.983** | 11.556 | **3.96E-23** | 817 |
|  |  | Breast (144) | Invasive Breast Carcinoma (76) | **1.621** | 9.386 | **1.17E-16** | 846 |
|  |  | Breast (144) | Invasive Lobular Breast Carcinoma (148) | **1.852** | 11.900 | **6.32E-27** | 1048 |
|  |  | Breast (144) | Invasive Ductal Breast Carcinoma (1,556) | **2.229** | 23.552 | **4.97E-58** | 1104 |
|  |  | Breast (144) | Ductal Breast Carcinoma in Situ (10) | **1.911** | 4.240 | **8.90E-4** | 1383 |
|  | TCGA Breast | Breast (61) | Invasive Ductal Breast Carcinoma (389) | **2.076** | 17.302 | **2.54E-32** | 485 |
|  |  | Breast (61) | Invasive Lobular Breast Carcinoma (36) | **1.616** | 7.551 | **6.56E-11** | 860 |
|  |  | Breast (61) | Invasive Breast Carcinoma (76) | **1.621** | 9.386 | **1.17E-16** | 905 |
|  |  | Breast (61) | Mixed Lobular and Ductal Breast Carcinoma (7) | **1.854** | 5.300 | **2.42E-4** | 1125 |
|  | Ma Breast 4 | Breast (14) | Ductal Breast Carcinoma in Situ (9) | **1.785** | 3.948 | **6.56E-4** | 794 |
| **OST4** | None | | | | | | |
| **STT3A** | Gluck Breast | Breast (4) | Invasive Breast Carcinoma (154) | **2.631** | 10.425 | **2.06E-4** | 687 |
|  | Finak Breast | Breast (6) | Invasive Breast Carcinoma (53) | **-14.771** | -16.800 | **7.48E-16** | 1870 |
| **STT3B** | Curtis Breast | Breast (144) | Mucinous Breast Carcinoma (46) | **1.608** | 9.667 | **2.59E-14** | 699 |
|  | Finak Breast | Breast (6) | Invasive Breast Carcinoma (53) | **-8.258** | -17.995 | **1.94E-25** | 622 |
| **DDOST** | None | | | | | | |
| **TUSC3** | Karnoub Breast | Breast (15) | Invasive Ductal Breast Carcinoma (7) | **-1.964** | -4.200 | **2.23E-4** | 358 |
|  | TCGA Breast | Breast (61) | Intraductal Cribriform Breast Adenocarcinoma (3) | **-2.195** | -8.733 | **1.03E-5** | 466 |
|  |  | Breast (61) | Invasive Breast Carcinoma (76) | **-2.210** | -8.875 | **2.18E-15** | 1192 |
|  |  | Breast (61) | Invasive Ductal Breast Carcinoma (389) | **-2.669** | -13.893 | **5.65E-24** | 1321 |
|  |  | Breast (61) | Invasive Lobular Breast Carcinoma (36) | **-1.974** | -6.318 | **9.72E-9** | 1782 |
|  | Finak Breast | Breast (6) | Invasive Breast Carcinoma (53) | **-19.212** | -18.771 | **5.63E-24** | 749 |
| **DAD1** | Zhao Breast | Breast (3) | Lobular Breast Carcinoma (21) | **2.112** | 12.275 | **6.48E-10** | 21 |
|  |  | Breast (3) | Invasive Ductal Breast Carcinoma (38) | **2.213** | 15.223 | **4.41E-10** | 172 |
|  | Curtis Breast | Breast (144) | Tubular Breast Carcinoma (67) | **1.512** | 13.473 | **3.13E-24** | 447 |
| **TMEM258** | None | | | | | | |
| **OSTC** | Curtis Breast | Breast (144) | Breast Carcinoma (14) | **1.651** | 9.095 | **1.34E-8** | 33 |
|  |  | Breast (144) | Ductal Breast Carcinoma in Situ (10) | **1.982** | 8.282 | **3.15E-6** | 54 |
|  |  | Breast (144) | Tubular Breast Carcinoma (67) | **1.760** | 11.052 | **2.73E-19** | 1007 |
| **KRTCAP2** | Ma Breast4 | Breast (14) | Invasive Ductal Breast Carcinoma (9) | **1.642** | 4.485 | **4.47E-4** | 560 |
|  | TCGA Breast | Breast (61) | Invasive Breast Carcinoma (76) | **1.567** | 8.876 | **1.87E-15** | 1075 |
|  |  | Breast (61) | Invasive Ductal Breast Carcinoma (389) | **1.512** | 10.861 | **8.80E-19** | 1817 |
| **MAGT1** | Curtis Breast | Breast (144) | Ductal Breast Carcinoma in Situ (10) | **-2.381** | -9.176 | **1.40E-6** | 429 |
|  |  | Breast (144) | Mucinous Breast Carcinoma (46) | **-2.247** | -14.057 | **9.31E-22** | 560 |
|  |  | Breast (144) | Invasive Ductal Breast Carcinoma (1,556) | **-1.852** | -22.591 | **2.78E-60** | 592 |
|  |  | Breast (144) | Tubular Breast Carcinoma (67) | **-2.313** | -15.079 | **1.33E-27** | 687 |
|  |  | Breast (144) | Invasive Lobular Breast Carcinoma (148) | **-1.744** | -11.897 | **2.71E-26** | 1142 |
|  |  | Breast (144) | Breast Carcinoma (14) | **-2.162** | -6.490 | **6.76E-6** | 1183 |
|  |  | Breast (144) | Invasive Ductal and Invasive Lobular Breast Carcinoma (90) | **-1.853** | -11.470 | **5.64E-22** | 1221 |
