# Supplementary material for: Knockdown of Oligosaccharyltransferase Subunit Ribophorin 1 Induces Endoplasmic-Reticulum-Stress-Dependent Cell Apoptosis in Breast Cancer
Source: Front Oncol. 2021 Oct 27;11:722624. doi: 10.3389/fonc.2021.722624 (PMC8578895; doi:10.3389/fonc.2021.722624)
Supplement: Supplementary file 11 [file Table_5.docx]

**Table S5** The relationship between the OST subunits and the clinicopathologic parameters of breast cancer (bc-GenExMiner v4.5 and v4.7)

|  |  | **RPN1** | | **RPN2** | | **OST4** | | **STT3A** | | **STT3B** | | **DDOST** | |
| --- | --- | --- | --- | --- | --- | --- | --- | --- | --- | --- | --- | --- | --- |
|  |  | Comp. | P | Comp. | P | Comp. | P | Comp. | P | Comp. | P | Comp. | P |
| Age | ≤ 51 |  | 0.8184 | ↑ | **0.0019** |  | 0.7755 |  | 0.1950 |  | 0.0578 |  | 0.4482 |
|  | > 51 |  |  |  |  |  |  |  |  |  |  |  |  |
| ER (IHC) | Negative | ↑ | **<0.0001** | ↑ | **0.0020** |  | 0.0101 | ↑ | **<0.0001** |  | 0.2391 | ↑ | **<0.0001** |
|  | Positive |  |  |  |  |  |  |  |  |  |  |  |  |
| PR (IHC) | Negative | ↑ | **<0.0001** | ↑ | **0.0003** | ↑ | **0.0044** |  | 0.1242 |  | 0.3620 | ↑ | **<0.0001** |
|  | Positive |  |  |  |  |  |  |  |  |  |  |  |  |
| HER2 (IHC) | Negative |  | 0.0661 | ↑ | **<0.0001** |  | 0.4899 |  | 0.1040 | ↑ | **<0.0001** |  | 0.0631 |
|  | Positive |  |  |  |  |  |  |  |  |  |  |  |  |
| Nodal status | Negative |  | 0.9624 |  | 0.1551 |  | **0.0004** | ↑ | **0.0289** |  | **<0.0001** |  | 0.5209 |
|  | Positive |  |  |  |  | ↑ |  |  |  | ↑ |  |  |  |
| Intrinsic subtype | Total |  | **<0.0001** |  | **<0.0001** |  | **<0.0001** |  | **<0.0001** |  | **<0.0001** |  | **<0.0001** |
| Basal-like vs Luminal A | | > | **<0.0001** | > | **<0.0001** | > | **<0.001** | > | **<0.0001** | > | **<0.0001** | > | **<0.0001** |
| Basal-like vs Luminal B | | > | **<0.0001** | < | **<0.0001** | = | >0.01 | > | **<0.0001** | < | **<0.0001** | > | **<0.0001** |
| Basal-like vs HER2-E | | > | **<0.05** | < | **<0.0001** | < | **<0.01** | = | >0.01 | < | **<0.0001** | > | **<0.0001** |
| Luminal B vs Luminal A | | > | **<0.0001** | > | **<0.0001** | > | **<0.0001** | < | **<0.001** | > | **<0.0001** | > | **<0.0001** |
| Luminal B vs HER2-E | | = | >0.01 | < | **<0.0001** | < | **<0.01** | < | **<0.0001** | < | **<0.001** | < | **<0.0001** |
| HER2-E vs Luminal A | | > | **<0.0001** | > | **<0.0001** | > | **<0.0001** | > | **<0.0001** | > | **<0.0001** | > | **<0.0001** |
| Basal-like status | Basal | ↑ | **<0.0001** |  | 0.4492 |  | 0.6746 | ↑ | **<0.0001** |  | 0.2047 | ↑ | **<0.0001** |
|  | None |  |  |  |  |  |  |  |  |  |  |  |  |
| SBR | Total |  | **<0.0001** |  | **<0.0001** |  | **<0.0001** |  | **0.0012** |  | **<0.0001** |  | **<0.0001** |
| SBR2 vs SBR1 | | > | **<0.0001** | > | **<0.0001** | > | **<0.01** | = | >0.01 | > | **<0.01** | > | **<0.01** |
| SBR3 vs SBR1 | | > | **<0.0001** | > | **<0.0001** | > | **<0.0001** | > | **<0.01** | > | **<0.0001** | > | **<0.0001** |
| SBR3 vs SBR2 | | > | **<0.0001** | > | **<0.0001** | > | **<0.05** | > | **<0.01** | > | **<0.01** | > | **<0.0001** |
| NPI | Total |  | **<0.0001** |  | **0.0015** |  | 0.0157 |  | 0.2014 |  | **0.0024** |  | 0.0715 |
| NPI2 vs NPI1 | | > | **<0.001** | > | **<0.01** | = | >0.01 | = | >0.01 | > | **<0.01** | = | >0.01 |
| NPI3 vs NPI1 | | > | **<0.001** | = | >0.01 | = | >0.01 | = | >0.01 | = | >0.01 | = | >0.01 |
| NPI3 vs NPI2 | | = | >0.01 | = | >0.01 | = | >0.01 | = | >0.01 | = | >0.01 | = | >0.01 |
|  |  | **TUSC3** | | **DAD1** | | **TMEM258** | | **OSTC** | | **KRTCAP2** | | **MAGT1** | |
|  |  | Comp. | P | Comp. | P | Comp. | P | Comp. | P | Comp. | P | Comp. | P |
| Age | ≤ 51 |  | **0.0012** |  | 0.1423 |  | 0.0412 |  | 0.0897 |  | 0.1187 |  | 0.9691 |
|  | > 51 | ↑ |  |  |  |  |  |  |  |  |  |  |  |
| ER (IHC) | Negative |  | 0.0112 |  | **<0.0001** | ↑ | **<0.0001** | ↑ | **0.0039** |  | 0.2756 | ↑ | **0.0027** |
|  | Positive |  |  | ↑ |  |  |  |  |  |  |  |  |  |
| PR (IHC) | Negative |  | 0.3271 |  | 0.1546 | ↑ | **<0.0001** |  | 0.7492 |  | 0.4137 |  | 0.3005 |
|  | Positive |  |  |  |  |  |  |  |  |  |  |  |  |
| HER2 (IHC) | Negative |  | 0.1967 |  | 0.1558 |  | 0.3758 |  | 0.0239 |  | 0.5041 |  | 0.4861 |
|  | Positive |  |  |  |  |  |  |  |  |  |  |  |  |
| Nodal status | Negative |  | 0.0465 |  | **0.0007** |  | 0.7373 |  | 0.0416 |  | 0.4352 |  | 0.1348 |
|  | Positive |  |  | ↑ |  |  |  |  |  |  |  |  |  |
| Intrinsic subtype | Total |  | **<0.0001** |  | **<0.0001** |  | **<0.0001** |  | **<0.0001** |  | **<0.0001** |  | **<0.0001** |
| Basal-like vs Luminal A | | > | **<0.0001** | < | **<0.0001** | > | **<0.0001** | > | **<0.0001** | > | **<0.0001** | = | >0.01 |
| Basal-like vs Luminal B | | > | **<0.0001** | < | **<0.0001** | > | **<0.0001** | > | **<0.0001** | = | >0.01 | = | >0.01 |
| Basal-like vs HER2-E | | > | **<0.0001** | < | **<0.0001** | = | >0.01 | = | >0.01 | = | >0.01 | = | >0.01 |
| Luminal B vs Luminal A | | < | **<0.0001** | > | **<0.0001** | > | **<0.0001** | > | **<0.0001** | > | **<0.0001** | > | **<0.01** |
| Luminal B vs HER2-E | | > | **<0.0001** | = | >0.01 | < | **<0.01** | = | >0.01 | = | >0.01 | = | >0.01 |
| HER2-E vs Luminal A | | < | **<0.0001** | > | **<0.001** | > | **<0.0001** | > | **<0.0001** | > | **<0.0001** | > | **<0.0001** |
| Basal-like status | Basal | ↑ | **<0.0001** |  | **<0.0001** | ↑ | **<0.0001** |  | 0.0634 | ↑ | **<0.0001** | ↑ | **0.005** |
|  | None |  |  | ↑ |  |  |  |  |  |  |  |  |  |
| SBR | Total |  | 0.3715 |  | 0.0389 |  | **<0.0001** |  | **<0.0001** |  | **0.0069** |  | **0.0005** |
| SBR2 vs SBR1 | |  |  | > | **<0.05** | > | **<0.0001** | > | **<0.001** | > | **<0.01** | = | >0.01 |
| SBR3 vs SBR1 | |  |  | = | >0.01 | > | **<0.0001** | > | **<0.0001** | = | >0.01 | > | **<0.01** |
| SBR3 vs SBR2 | |  |  | = | >0.01 | > | **<0.0001** | = | >0.01 | = | >0.01 | > | **<0.01** |
| NPI |  |  | 0.8025 |  | 0.0699 |  | **<0.0001** |  | 0.0894 |  | 0.3639 |  | 0.3223 |
| NPI2 vs NPI1 | | = | >0.01 | = | >0.01 | > | **<0.0001** | = | >0.01 | = | >0.01 | = | >0.01 |
| NPI3 vs NPI1 | | = | >0.01 | = | >0.01 | > | **<0.01** | = | >0.01 | = | >0.01 | = | >0.01 |
| NPI3 vs NPI2 | | = | >0.01 | = | >0.01 | = | >0.01 | = | >0.01 | = | >0.01 | = | >0.01 |

**Notes:** The data with statistical significance (P<0.01) were marked in bold text. In addition, the data of intrinsic subtype was obtained from bc-GenExMiner v4.7 due to the update of the website.

**Abbreviation:** Comp, comparison; IHC, immunohistochemical; ER, estrogen receptor; PR, progesterone receptor; HER2, human epidermal growth factor receptor 2; HER2-E, HER2-enriched; SBR, Scarff Bloom & Richardson grade; NPI, Nottingham Prognostic Index.
