# Supplementary material for: Knockdown of Oligosaccharyltransferase Subunit Ribophorin 1 Induces Endoplasmic-Reticulum-Stress-Dependent Cell Apoptosis in Breast Cancer
Source: Front Oncol. 2021 Oct 27;11:722624. doi: 10.3389/fonc.2021.722624 (PMC8578895; doi:10.3389/fonc.2021.722624)
Supplement: Supplementary file 12 [file Table_6.docx]

**Table S6** Survival analyses of the OST subunits in all breast cancer.

| **Gene** | **Affymetrix ID** | **Survival outcome** | **Number of patients** | **HR** | **95%CI** | **logrank P** |
| --- | --- | --- | --- | --- | --- | --- |
| RPN1 | 201011_at | RFS | 3951 | 1.51 | 1.35-1.69 | **1.2E-13** |
|  |  | OS | 1402 | 1.35 | 1.09-1.68 | **0.006** |
|  |  | DMFS | 1746 | 1.2 | 0.98-1.45 | 0.07 |
|  |  | PPS | 414 | 1.17 | 0.91-1.49 | 0.22 |
| RPN2 | 213491_x_at | RFS | 3951 | 1.26 | 1.13-1.4 | **3.6E-05** |
|  |  | OS | 1402 | 1.49 | 1.2-1.85 | **0.00031** |
|  |  | DMFS | 1746 | 1.18 | 0.97-1.43 | 0.098 |
|  |  | PPS | 414 | 0.93 | 0.73-1.19 | 0.57 |
| OST4 | 224637_at | RFS | 1764 | 1.56 | 1.34-1.83 | **1.9E-08** |
|  |  | OS | 626 | 0.98 | 0.72-1.34 | 0.91 |
|  |  | DMFS | 664 | 0.84 | 0.61-1.17 | 0.31 |
|  |  | PPS | 173 | 0.83 | 0.58-1.18 | 0.3 |
| STT3A | 202223_at | RFS | 3951 | 1.15 | 1.03-1.28 | **0.013** |
|  |  | OS | 1402 | 0.92 | 0.75-1.15 | 0.47 |
|  |  | DMFS | 1746 | 0.93 | 0.76-1.12 | 0.44 |
|  |  | PPS | 414 | 0.85 | 0.66-1.08 | 0.18 |
| STT3B | 224700_at | RFS | 1764 | 1.13 | 0.97-1.32 | 0.11 |
|  |  | OS | 626 | 0.76 | 0.55-1.03 | 0.08 |
|  |  | DMFS | 664 | 1.51 | 1.08-2.1 | **0.014** |
|  |  | PPS | 173 | 0.74 | 0.52-1.07 | 0.11 |
| DDOST | 208675_s_at | RFS | 3951 | 0.98 | 0.88-1.09 | 0.69 |
|  |  | OS | 1402 | 0.8 | 0.65-1 | **0.045** |
|  |  | DMFS | 1746 | 0.75 | 0.61-0.91 | **0.0031** |
|  |  | PPS | 414 | 0.8 | 0.63-1.03 | 0.078 |
| TUSC3 | 200046_at | RFS | 1764 | 0.72 | 0.62-0.84 | **4E-05** |
|  |  | OS | 626 | 0.94 | 0.69-1.28 | 0.68 |
|  |  | DMFS | 664 | 0.84 | 0.61-1.16 | 0.3 |
|  |  | PPS | 173 | 0.94 | 0.66-1.34 | 0.75 |
| DAD1 | 218213_s_at | RFS | 3951 | 1.25 | 1.12-1.39 | **7.7E-05** |
|  |  | OS | 1402 | 1.14 | 0.92-1.41 | 0.24 |
|  |  | DMFS | 1746 | 1.01 | 0.83-1.23 | 0.9 |
|  |  | PPS | 414 | 0.81 | 0.64-1.04 | 0.098 |
| OSTC | 223001_at | RFS | 1764 | 1.46 | 1.25-1.71 | **1.7E-06** |
|  |  | OS | 626 | 1.00 | 0.73-1.37 | 1 |
|  |  | DMFS | 664 | 1.08 | 0.78-1.49 | 0.65 |
|  |  | PPS | 173 | 0.85 | 0.60-1.21 | 0.37 |
| TMEM258 | 218213_s_at | RFS | 3951 | 1.28 | 1.15-1.43 | **6.6E-06** |
|  |  | OS | 1402 | 1.22 | 0.98-1.51 | 0.07 |
|  |  | DMFS | 1746 | 1.04 | 0.85-1.26 | 0.72 |
|  |  | PPS | 414 | 0.82 | 0.65-1.05 | 0.12 |
| KRTGAP2 | 224885_s_at | RFS | 1764 | 1.34 | 1.15-1.57 | **0.00019** |
|  |  | OS | 626 | 1.19 | 0.87-1.63 | 0.27 |
|  |  | DMFS | 664 | 0.97 | 0.7-1.35 | 0.87 |
|  |  | PPS | 173 | 0.9 | 0.64-1.29 | 0.58 |
| MAGT1 | 224899_s_at | RFS | 1764 | 1.09 | 0.93-1.27 | 0.27 |
|  |  | OS | 626 | 0.92 | 0.67-1.26 | 0.61 |
|  |  | DMFS | 664 | 1.04 | 0.76-1.44 | 0.79 |
|  |  | PPS | 173 | 0.58 | 0.4-0.83 | **0.0026** |
