# Supplementary material for: Knockdown of Oligosaccharyltransferase Subunit Ribophorin 1 Induces Endoplasmic-Reticulum-Stress-Dependent Cell Apoptosis in Breast Cancer
Source: Front Oncol. 2021 Oct 27;11:722624. doi: 10.3389/fonc.2021.722624 (PMC8578895; doi:10.3389/fonc.2021.722624)
Supplement: Supplementary file 13 [file Table_7.docx]

**Table S7** Survival analyses of the OST subunits with different molecular subtypes in breast cancer

|  |  |  | Basal-like | | | Luminal A | | | Luminal B | | | HER2-enriched | | |
| --- | --- | --- | --- | --- | --- | --- | --- | --- | --- | --- | --- | --- | --- | --- |
| Gene | Affymetrix ID | Survival outcome | Num of patients | HR (95%CI) | logrank P | Num of patients | HR (95%CI) | logrank P | Num of patients | HR (95%CI) | logrank P | Num of patients | HR (95%CI) | logrank P |
| RPN1 | 201011_at | RFS | 618 | 1.31 (1.01-1.68) | **0.038** | 1933 | 1.38 (1.16-1.64) | **0.00024** | 1149 | 1.24 (1.03-1.51) | **0.025** | 251 | 1.67 (1.13-2.47) | **0.0093** |
|  |  | OS | 241 | 0.67 (0.41-1.1) | 0.11 | 611 | 1.37 (0.96-1.96) | 0.083 | 433 | 1.32 (0.91-1.93) | 0.14 | 117 | 1.4 (0.73-2.68) | 0.31 |
|  |  | DMFS | 232 | 1.19 (0.71-1.98) | 0.51 | 965 | 1.11 (0.83-1.48) | 0.47 | 430 | 1.08 (0.76-1.53) | 0.68 | 119 | 1.42 (0.76-2.65) | 0.27 |
|  |  | PPS | 64 | 0.46 (0.26-0.84) | **0.0088** | 179 | 1.01 (0.68-1.49) | 0.97 | 134 | 0.96 (0.62-1.47) | 0.85 | 37 | 0.72 (0.34-1.52) | 0.39 |
| RPN2 | 213491_x_at | RFS | 618 | 0.87 (0.67-1.12) | 0.27 | 1933 | 1.54 (1.29-1.83) | **9.1E-07** | 1149 | 1.12 (0.92-1.35) | 0.26 | 251 | 1.2 (0.82-1.77) | 0.35 |
|  |  | OS | 241 | 0.91 (0.55-1.48) | 0.69 | 611 | 1.53 (1.07-2.19) | **0.019** | 433 | 1.5 (1.03-2.19) | **0.034** | 117 | 1.43 (0.74-2.76) | 0.28 |
|  |  | DMFS | 232 | 0.83 (0.5-1.38) | 0.46 | 965 | 1.04 (078-1.39) | 0.78 | 430 | 1.44 (1.01-2.05) | **0.044** | 119 | 0.67 (0.36-1.26) | 0.21 |
|  |  | PPS | 64 | 0.89 (0.5-1.59) | 0.69 | 179 | 1.07 (0.73-1.58) | 0.72 | 134 | 0.78 (0.51-1.21) | 0.27 | 37 | 0.71 (0.34-1.5) | 0.37 |
| OST4 | 224637_at | RFS | 360 | 1.8 (1.3-2.51) | **0.00038** | 841 | 1.47 (1.15-1.89) | **0.0023** | 407 | 1.77 (1.3-2.42) | **0.00028** | 156 | 1.25 (0.79-1.97) | 0.34 |
|  |  | OS | 153 | 1.13 (0.6-2.14) | 0.7 | 271 | 0.88 (0.53-1.47) | 0.63 | 129 | 0.82 (0.42-1.6) | 0.55 | 73 | 1.19 (0.54-2.63) | 0.66 |
|  |  | DMFS | 145 | 1.3 (0.64-2.64) | 0.47 | 281 | 0.66 (0.38-1.15) | 0.14 | 156 | 0.84 (0.43-1.61) | 0.59 | 82 | 0.85 (0.4-1.78) | 0.66 |
|  |  | PPS | 33 | 0.52 (0.22-1.22) | 0.13 | 75 | 0.97 (0.55-1.71) | 0.91 | 37 | 0.53 (0.25-1.11) | 0.087 | 28 | 1.07 (0.46-2.47) | 0.88 |
| STT3A | 202223_at | RFS | 618 | 1.42 (1.1-1.83) | **0.0063** | 1933 | 0.88 (0.74-1.04) | 0.14 | 1149 | 1.24 (1.02-1.5) | **0.028** | 251 | 1.21 (0.82-1.78) | 0.33 |
|  |  | OS | 241 | 0.66 (0.4-1.08) | 0.097 | 611 | 0.74 (0.52-1.06) | 0.1 | 433 | 0.87 (0.6-1.26) | 0.45 | 117 | 0.62 (0.32-1.19) | 0.15 |
|  |  | DMFS | 232 | 1.06 (0.64-1.75) | 0.83 | 965 | 0.72 (0.54-0.96) | **0.026** | 430 | 1.17 (0.82-1.67) | 0.37 | 119 | 0.79 (0.42-1.47) | 0.45 |
|  |  | PPS | 64 | 0.67 (0.37-1.21) | 0.18 | 179 | 0.83 (0.56-1.22) | 0.34 | 134 | 0.79 (0.52-1.22) | 0.29 | 37 | 0.51 (0.24-1.11) | 0.084 |
| STT3B | 224700_at | RFS | 360 | 1.24 (0.9-1.72) | 0.19 | 831 | 1.12 (0.87-1.43) | 0.38 | 407 | 1.23 (0.91-1.68) | 0.18 | 156 | 0.75 (0.48-1.18) | 0.21 |
|  |  | OS | 153 | 0.85 (0.45-1.62) | 0.63 | 271 | 0.91 (0.55-1.51) | 0.71 | 129 | 0.92(0.47-1.8) | 0.8 | 73 | 0.56 (0.25-1.26) | 0.16 |
|  |  | DMFS | 145 | 1.64 (0.79-3.37) | 0.18 | 281 | 2.09 (1.17-3.73) | **0.011** | 156 | 1.38 (0.71-2.68) | 0.34 | 82 | 0.62 (0.29-1.31) | 0.2 |
|  |  | PPS | 33 | 0.95 (0.41-2.15) | 0.89 | 75 | 0.86 (0.48-1.52) | 0.6 | 37 | 0.57 (0.27-1.19) | 0.13 | 28 | 0.56 (0.24-1.31) | 0.18 |
| DDOST | 208675_s_at | RFS | 618 | 0.94 (0.73-1.21) | 0.65 | 1933 | 0.93 (0.79-1.11) | 0.42 | 1149 | 1 (0.82-1.21) | 0.98 | 251 | 0.68 (0.46-1) | **0.049** |
|  |  | OS | 241 | 0.69 (0.42-1.13) | 0.14 | 611 | 0.64 (0.45-0.92) | **0.015** | 433 | 1.08 (0.74-1.57) | 0.7 | 117 | 0.55 (0.28-1.06) | 0.07 |
|  |  | DMFS | 232 | 1.02 (0.62-1.7) | 0.93 | 965 | 0.71 (0.53-0.94) | **0.018** | 430 | 0.86 90.6-1.22) | 0.39 | 119 | 0.64 (0.34-1.21) | 0.17 |
|  |  | PPS | 64 | 0.8 (0.44-1.43) | 0.45 | 179 | 0.78 (0.53-1.15) | 0.21 | 134 | 1.02 (0.67-1.57) | 0.91 | 37 | 0.63 (0.3-1.35) | 0.23 |
| TUSC3 | 235801_at | RFS | 360 | 1 (0.73-1.38) | 0.99 | 841 | 0.74 (0.58-0.95) | **0.016** | 407 | 0.84 (0.62-1.14) | 0.26 | 156 | 0.96 (0.61-1.51) | 0.86 |
|  |  | OS | 153 | 1.11 (0.59-2.09) | 0.75 | 271 | 0.86 (0.52-1.42) | 0.55 | 129 | 1.41 (0.71-2.8) | 0.32 | 73 | 0.5 (0.22-1.12) | 0.086 |
|  |  | DMFS | 145 | 1 (0.49-2.02) | 0.99 | 281 | 1.14 (0.66-1.97) | 0.64 | 156 | 0.61 (0.31-1.19) | 0.14 | 82 | 1.07 (0.51-2.26) | 0.86 |
|  |  | PPS | 33 | 1.32 (0.57-3.08) | 0.51 | 75 | 0.81 (0.46-1.41) | 0.45 | 37 | 1.09 (0.52-2.3) | 0.81 | 28 | 1.31 (0.56-3.09) | 0.54 |
| DAD1 | 200046_at | RFS | 618 | 1.39 (1.08-1.8) | **0.0096** | 1933 | 1.19 (1-1.41) | **0.044** | 1149 | 1.27 (1.05-1.54) | **0.013** | 251 | 1.51 (1.03-2.22) | **0.036** |
|  |  | OS | 241 | 1.1 (0.67-1.79) | 0.72 | 611 | 1.04 (0.73-1.48) | 0.82 | 433 | 1.32 (0.91-1.92) | 0.14 | 117 | 1.17 (0.61-2.24) | 0.63 |
|  |  | DMFS | 232 | 1.36 (0.82-2.27) | 0.23 | 965 | 0.85 (0.64-1.13) | 0.27 | 430 | 1.27 (0.89-1.8) | 0.19 | 119 | 1.11 (0.6-2.07) | 0.74 |
|  |  | PPS | 64 | 0.61 (0.33-1.1) | 0.096 | 179 | 0.83 (0.56-1.23) | 0.36 | 134 | 0.83 (0.54-1.28) | 0.4 | 37 | 0.66 (0.31-1.4) | 0.27 |
| TMEM258 | 218213_s_at | RFS | 618 | 0.75 (0.58-0.96) | **0.023** | 1933 | 1.42 (1.19-1.68) | **6.5E-05** | 1149 | 1.26 (1.04-1.53) | **0.016** | 251 | 0.9 (0.61-1.32) | 0.59 |
|  |  | OS | 241 | 0.62 (0.37-1.02) | 0.058 | 611 | 1.04 (0.73-1.48) | 0.82 | 433 | 1.29 (0.89-1.86) | 0.18 | 117 | 1.41 (0.73-2.69) | 0.3 |
|  |  | DMFS | 232 | 0.53 (0.31-0.9) | **0.018** | 965 | 0.93 (0.7-1.24) | 0.63 | 430 | 1.11 (0.78-1.57) | 0.57 | 119 | 0.81 (0.43-1.52) | 0.51 |
|  |  | PPS | 64 | 0.61 (0.33-1.11) | 0.1 | 179 | 0.83 (0.56-1.23) | 0.35 | 134 | 0.78 (0.51-1.19) | 0.24 | 37 | 0.79 (0.37-1.68) | 0.55 |
| OSTC | 223001_at | RFS | 360 | 1.35 (0.98-1.87) | 0.067 | 841 | 1.48 (1.16-1.90) | **0.0018** | 407 | 1.48 (1.09-2.02) | **0.011** | 156 | 0.87 (0.55-1.37) | 0.54 |
|  |  | OS | 153 | 1.02 (0.54-1.94) | 0.94 | 271 | 0.88 (0.53-1.56) | 0.62 | 129 | 1.23 (0.62-2.42) | 0.56 | 73 | 0.42 (0.18-0.97) | **0.037** |
|  |  | DMFS | 145 | 2.03 (0.97-4.25) | 0.055 | 281 | 0.82 (0.47-1.41) | 0.46 | 156 | 0.98 (0.51-1.89) | 0.96 | 82 | 0.51 (0.23-1.10) | 0.079 |
|  |  | PPS | 33 | 0.79 (0.35-1.80) | 0.57 | 75 | 0.63 (0.36-1.13) | 0.12 | 37 | 1.01 (0.49-2.11) | 0.97 | 28 | 0.60 (0.25-1.42) | 0.24 |
| KRTGAP2 | 224885_s_at | RFS | 360 | 1.59 (1.15-2.21) | **0.0047** | 841 | 1.27 (0.99-1.63) | 0.055 | 407 | 1.28 (0.94-1.73) | 0.12 | 156 | 1.08 (0.69-1.71) | 0.72 |
|  |  | OS | 153 | 1.16 (0.61-2.19) | 0.65 | 271 | 1.16 (0.7-1.94) | 0.56 | 129 | 0.91 (0.46-1.79) | 0.79 | 73 | 3.28(1.37-7.88) | **0.0049** |
|  |  | DMFS | 145 | 1.5 (0.73-3.07) | 0.26 | 281 | 0.81 (0.47-1.4) | 0.45 | 156 | 1.24 (0.64-2.4) | 0.52 | 82 | 0.82 (0.39-1.73) | 0.6 |
|  |  | PPS | 33 | 1.31 (0.56-3.02) | 0.53 | 75 | 0.81 (0.46-1.42) | 0.47 | 37 | 0.63 (0.3-1.31) | 0.21 | 28 | 1.62 (0.7-3.77) | 0.26 |
| MAGT1 | 224899_s_at | RFS | 360 | 1.17 (0.85-1.62) | 0.34 | 841 | 1 (0.78-1.28) | 0.98 | 407 | 1.16 (0.85-1.57) | 0.35 | 156 | 0.8 (0.51-1.27) | 0.34 |
|  |  | OS | 153 | 1.24 (0.65-2.35) | 0.52 | 271 | 0.65(0.39-1.09) | 0.1 | 129 | 1.39 (0.7-2.74) | 0.34 | 73 | 0.42 (0.18-0.97) | **0.035** |
|  |  | DMFS | 145 | 1.86 (0.89-3.86) | 0.092 | 281 | 0.67 (0.39-1.17) | 0.15 | 156 | 1.19 (0.62-2.3) | 0.6 | 82 | 0.73 (0.34-1.54) | 0.4 |
|  |  | PPS | 33 | 0.78 (0.33-1.85) | 0.57 | 75 | 0.41 (0.23-0.75) | **0.0031** | 37 | 0.82 (0.39-1.7) | 0.59 | 28 | 0.42 (0.17-1) | **0.044** |

**Notes:** The molecular subtypes were based on the 2013 St Gallen criteria. All of the data above were obtained from the Kaplan-Meier Plotter database.

The data with statistical significance (P<0.05) were marked in bold text.

**Abbreviation:** HR, hazard ratio; CI, confidence interval; OS, overall survival; RFS, relapse free survival; DMFS, distant metastasis free survival; PPS, post progression survival.
