# Supplementary material for: Knockdown of Oligosaccharyltransferase Subunit Ribophorin 1 Induces Endoplasmic-Reticulum-Stress-Dependent Cell Apoptosis in Breast Cancer
Source: Front Oncol. 2021 Oct 27;11:722624. doi: 10.3389/fonc.2021.722624 (PMC8578895; doi:10.3389/fonc.2021.722624)
Supplement: Supplementary file 14 [file Table_8.docx]

**Table S8** The details of the IHC figures of RNN1 in the HPA

|  |  | Normal-1 | Normal-2 | Tumor-1 | Tumor-2 |
| --- | --- | --- | --- | --- | --- |
| Patient’ information | Age | 23 | 27 | 41 | 59 |
|  | Tissue Type | Normal tissue | Normal tissue | Lobular carcinoma, in situ | Lobular carcinoma |
|  | Patient ID | 2773 | 3286 | 2898 | 2805 |
| IHC | Staining | Low | Low | Medium | Medium |
|  | Intensity | Moderate | Moderate | Moderate | Moderate |
|  | Quantity | <25% | <25% | 75%-25% | >75% |

**Abbreviation:** IHC, Immunohistochemistry
