# Supplementary material for: Knockdown of Oligosaccharyltransferase Subunit Ribophorin 1 Induces Endoplasmic-Reticulum-Stress-Dependent Cell Apoptosis in Breast Cancer
Source: Front Oncol. 2021 Oct 27;11:722624. doi: 10.3389/fonc.2021.722624 (PMC8578895; doi:10.3389/fonc.2021.722624)
Supplement: Supplementary file 15 [file Table_9.docx]

**Table S9** A total of 46 genes positively co-expressed with RPN1 (bc-GenExMiner v4.5).

| Number | Gene symbol | Pearson's correlation coefficient | p-value | No. patients |
| --- | --- | --- | --- | --- |
| 1 | SEC61A1 | 0.7511 | < 0.0001 | 317 |
| 2 | COPG1 | 0.7127 | < 0.0001 | 317 |
| 3 | SRPRB | 0.6928 | < 0.0001 | 317 |
| 4 | RAB7A | 0.6375 | < 0.0001 | 317 |
| 5 | MRPL3 | 0.5586 | < 0.0001 | 317 |
| 6 | ACAD9 | 0.5528 | < 0.0001 | 317 |
| 7 | COPB2 | 0.5457 | < 0.0001 | 317 |
| 8 | HMCES | 0.5368 | < 0.0001 | 317 |
| 9 | RNF7 | 0.534 | < 0.0001 | 317 |
| 10 | MBD4 | 0.5214 | < 0.0001 | 317 |
| 11 | TPRA1 | 0.5201 | < 0.0001 | 317 |
| 12 | MRPS22 | 0.51 | < 0.0001 | 317 |
| 13 | TIMMDC1 | 0.5031 | < 0.0001 | 317 |
| 14 | PDIA5 | 0.501 | < 0.0001 | 317 |
| 15 | RUVBL1 | 0.4977 | < 0.0001 | 317 |
| 16 | ATP2C1 | 0.4856 | < 0.0001 | 317 |
| 17 | CALR | 0.4822 | < 0.0001 | 317 |
| 18 | NDUFB4 | 0.4734 | < 0.0001 | 317 |
| 19 | SEC13 | 0.4728 | < 0.0001 | 317 |
| 20 | TFG | 0.4714 | < 0.0001 | 317 |
| 21 | EEFSEC | 0.4685 | < 0.0001 | 317 |
| 22 | CNBP | 0.4642 | < 0.0001 | 317 |
| 23 | ATP6V0B | 0.4585 | < 0.0001 | 317 |
| 24 | EBP | 0.4579 | < 0.0001 | 317 |
| 25 | ISY1 | 0.4515 | < 0.0001 | 317 |
| 26 | DNAJB11 | 0.45 | < 0.0001 | 317 |
| 27 | ALG3 | 0.4457 | < 0.0001 | 317 |
| 28 | SERP1 | 0.4433 | < 0.0001 | 317 |
| 29 | PCCB | 0.4317 | < 0.0001 | 317 |
| 30 | SEC22A | 0.4297 | < 0.0001 | 317 |
| 31 | RAB5IF | 0.4295 | < 0.0001 | 317 |
| 32 | DDOST | 0.4261 | < 0.0001 | 317 |
| 33 | MED8 | 0.4254 | < 0.0001 | 317 |
| 34 | ATP1B3 | 0.4248 | < 0.0001 | 317 |
| 35 | MRPS15 | 0.4165 | < 0.0001 | 317 |
| 36 | LRRC59 | 0.4152 | < 0.0001 | 317 |
| 37 | ECE2 | 0.412 | < 0.0001 | 317 |
| 38 | PRDX1 | 0.4116 | < 0.0001 | 317 |
| 39 | UBA5 | 0.4093 | < 0.0001 | 317 |
| 40 | FAM162A | 0.4083 | < 0.0001 | 317 |
| 41 | PPIB | 0.4081 | < 0.0001 | 317 |
| 42 | COX17 | 0.407 | < 0.0001 | 317 |
| 43 | TK1 | 0.4066 | < 0.0001 | 317 |
| 44 | CCDC58 | 0.4052 | < 0.0001 | 317 |
| 45 | BOLA3 | 0.4031 | < 0.0001 | 317 |
| 46 | GMPS | 0.4028 | < 0.0001 | 317 |
